# Extensive mobilome heterogeneity in a widespread endosymbiont: A candidate dimeric plasmid and highly fragmented prophages in *Wolbachia* from *Culex pipiens*

**DOI:** 10.1371/journal.pgen.1012347

**Published:** 2026-10-26

**Authors:** Alice Brunner, Maxime Mahout, Julien Amoros, Massilva Rahmoun, Mateo Jarry, Seth R. Bordenstein, Sarah R. Bordenstein, Blandine Trouche, Julie Reveillaud

**Affiliations:** 1 MIVEGEC, University of Montpellier, INRAe, CNRS, IRD, Montpellier, France; 2 Departments of Biology and Entomology, One Health Microbiome Center, Huck Institutes of the Life Sciences, Pennsylvania State University, University Park, Pennsylvania, United States of America; University of California, Los Angeles, UNITED STATES OF AMERICA

## Abstract

The obligate, intracellular bacteria *Wolbachia* have gained increasing interest due to their selfish modifications of host arthropod reproduction, impacts on host evolution, and utility in vector control efforts to reduce arbovirus transmission worldwide. Despite their highly reduced genomes, *Wolbachia* harbor a rich mobilome that includes phages, insertion sequences and plasmids in mosquito vectors. However, these repeat-rich mobile genetic elements are notoriously difficult to assemble and can generate artificial, chimeric sequences that obscure their true diversity. As a result, conventional genome sequencing and assembly approaches often fail to resolve the complexity, leaving critical gaps of knowledge on how these elements evolve, mobilize, and shape both symbiont and host genomes. To address these challenges, we established a novel Oxford Nanopore long-read sequencing strategy that avoids prior PCR amplification and leverages single-dissected, female ovaries. Using this approach, we report and characterize—directly from native long-read molecules—the first near-complete *Wolbachia* genome and its mobile elements from individual ovaries of the mosquito vector *Culex pipiens molestus*. Additional short reads from individuals of the same strain were used to assess and validate candidate mutations, particularly in repetitive regions. We report a new dimeric structure of the pWCP plasmid, suggesting the existence of multimeric plasmid forms potentially associated with active replication. We also observed extensive fragmentation of prophage WO regions despite long read sequencing, underscoring their structural complexity. Raw long read analyses recovered multiple alternative gene syntenies within WO regions, pointing to heterogeneous prophage architectures missed by the assembly and marked diversity of WO elements in *Wolbachia* of *Culex pipiens* (*w*Pip) group strains. Taken together, our results illuminate the structural heterogeneity of the endosymbiont genome that is shaped by integrated and episomal mobile elements.

## Author summary

Intracellular bacteria that live within the cells of animals are widespread and can profoundly influence their hosts’ biology. Among them, *Wolbachia* are one of the most influential and extensively studied. It occurs in numerous insect species, manipulates host reproduction, reduces viral replication, and is widely used in mosquito control programs. A hallmark of intracellular bacteria such as *Wolbachia* is the abundance of mobile genetic elements in their genomes, notably prophage-derived regions. These elements are major contributors to genome evolution and functional innovation. However, they are highly variable and notoriously difficult to assemble accurately, particularly when DNA amplification introduces artificial rearrangements. To better resolve this complexity, we developed a long-range sequencing approach that avoids prior PCR amplification and applied it at the scale of a single mosquito ovary. Using this strategy, we generated a near-complete *Wolbachia* genome and uncovered unexpected structural variation in its mobile elements. Notably, we provide evidence for a previously unrecognized dimeric structure of the pWCP *Wolbachia* plasmid, that could indicate active replication, as well as multiple alternative phage-associated configurations. Together, these findings reveal hidden genomic diversity and underscore the dynamic nature of intracellular bacterial genomes, despite the constraints imposed by life within host cells.

## Introduction

*Wolbachia* are a genus of obligate, intracellular α-proteobacteria (Rickettsiales) that occur in an estimated half of all arthropod species [1], including numerous vectors of infectious diseases, as well as most pathogenic filarial nematodes [2]. It is the most common bacterial endosymbiont in animals [3,4]. Primarily transmitted through the maternal germline, *Wolbachia* are renowned for their remarkable ability to manipulate host reproduction in multiple ways, including feminization, parthenogenesis, male killing and the most common — cytoplasmic incompatibility (CI) — a selfish phenomenon that causes embryonic death in crosses between infected males and uninfected females, or between individuals carrying distinct, incompatible *Wolbachia* strains [3,5]. In addition to these reproductive effects, *Wolbachia* modulate host susceptibility to pathogens, a feature that has sparked intense interest in their use as a biological tool to control mosquito-borne diseases such as dengue, chikungunya, or West Nile virus [6]. While vertical, transovarial transmission from mothers to offspring remains the dominant mode of inheritance, phylogenetic incongruities between host and symbiont lineages indicate that horizontal transfer events occurred during evolution, contributing to the global spread of *Wolbachia* [7,8].

A distinguishing driver of *Wolbachia* evolution and genome plasticity is its mobilome, the set of mobile genetic elements (MGEs) present in its genome or cytoplasm. These elements include phages, plasmids, and small MGEs such as insertion sequences and transposons [7,9–12]. They can occur in high frequency within some *Wolbachia*, constituting up to 38% of their genomes [8,13]. Among these elements, temperate bacteriophage WO or formally “*Wovirus*” is the best studied [12]. It commonly occurs as an intact or relic prophage in several *Wolbachia* supergroups, including A, B, E, F, and S [14]. WO typically carries a conserved set of core genes involved in functions such as capsid and tail morphogenesis, lysis, and lysogeny [12,15]. In addition, most WO phages harbor a unique eukaryotic association module (EAM), an accessory region which encodes some proteins with domains from eukaryotic origin, implicating these modules in varied functions of the *Wolbachia* – insect symbiosis [12,16]. These modules often include large numbers of ankyrin repeat proteins that are highly duplicated and potentially involved in mediating *Wolbachia* - host interactions [16–18]. In addition, EAM regions harbor genes underpinning reproductive manipulations, including *wmk* and *oscar* in male killing, *pifA- pifB* in parthenogenesis, and *cif*A-*cif*B in CI [19–22]. The latter code for proteins with varying domain and architectures classified into five [5] and tentatively up to ten phylogenetic types [23,24], with at least type I, II, and IV demonstrated as CI effectors [20,21,25].

Less studied and recently discovered, plasmids are another component of the *Wolbachia* mobilome. Initially detected in *Wolbachia* from *Culex pipiens* [9] and subsequently in *Aedes albopictus* [10], these extrachromosomal elements remain largely unexplored. In *Culex pipiens*, the plasmid pWCP is highly conserved across distant populations and localities worldwide [26], suggesting a recent spread and/or strong purifying selection. A fairly stable copy number per *Wolbachia* cell also suggests there may be functional consequences with the bacterium during most developmental stages of the mosquito host [27]. However, it is still considered putative due to the absence of a clearly defined origin of replication, and our understanding of the biology and inheritance of the element is still in its early stages.

Recent large-scale genome studies shed light on the sequence diversity of *Wolbachia* [28]. Within insect hosts, *Culex* mosquitoes stand out since more than 99% of species in this genus are naturally infected [29], and lineages of the *Culex pipiens* complex have long served as a reference system for investigating CI and vector control [30–33]. This genus is notable for the diversification of its *Wolbachia* strains into five *w*Pip phylogenetic groups, named *w*Pip-I to -V, which are directly correlated with CI cross compatibility [29,34]. To date, only three draft genomes are surprisingly available for *Wolbachia* infecting *Culex* mosquitoes: *w*PipPel (from *Culex quinquefasciatus* Pel) [35], *w*PipJHB (from *Culex quinquefasciatus* JHB) [36], and *w*PipMol (from *Culex pipiens molestus*) [37]. The most studied and conventionally used reference, *w*PipPel, was assembled from pools of embryos, but it is reported as a chromosome with assembly gaps, likely due to complex prophage regions and potential phage activity [35]. Subsequent analyses revealed that key loci such as the *cifA-cifB* (phylogenetic Type I) are present in multiple copies in individual *Culex pipiens* specimens [32], suggesting that the single copy reported in *w*PipPel may result from assembly collapse in repeated regions [32]. The second genome, *w*PipJHB, displayed major structural differences compared to *w*PipPel, with large rearrangements and several highly similar contigs discarded, potentially masking strain-level variation [36]. The third genome, *w*PipMol, remained fragmented despite using a different adult sample source [37]. Therefore, while the available *w*Pip genomes in *Culex* spp. are useful references, they remain consensus, fragmented assemblies that limit investigation of the fine-scale architecture, function, and genomic variation of *Wolbachia*, as well as the breadth and dynamics of its MGE, which are likely underestimated. Short read sequencing of single ovaries from four *Culex pipiens* specimens highlighted high phage variation between individuals together with the presence of fragmented contigs belonging to a novel MGE [9]. The fragmented nature of the four *Wolbachia* Metagenome-Assembled Genomes (MAGs) was not sufficient to resolve key genomic regions, and long read sequencing was necessary to confirm the whole circular pWCP plasmid element. Overall, the presence of numerous repeated regions in *Wolbachia* genomes in *Culex* lead to fragmented assemblies and achieving a closed and circular *Wolbachia* reference genome remains challenging [9,35,38,39].

Here, we reconstruct and analyze a new *Wolbachia* genome obtained from long read sequencing of individual ovaries from a gravid *Culex pipiens molestus* specimen. First, we developed an *in-house* protocol leveraging the sequencing capacity of MinION technology (ONT) without any upstream nucleic acid amplification in order to reduce the introduction of possible PCR errors and chimeric sequences between highly repeated genomic regions. In addition, we integrated independent Illumina-sequenced ovary metagenomes to verify specific point mutations. Second, a fine-scale analysis of raw long reads and coverage data showed a higher number and different organization of WO phage modules compared to previously assembled *w*Pip genomes and two forms of the plasmids, including a putative dimeric one, suggesting pWCP may represent a self-replicative element within a highly dynamic mobilome.

## Results

### Ovaries with high *Wolbachia* abundance were selected for genome sequencing

To maximize the likelihood of assembling a complete *Wolbachia* genome, we first evaluated the bacterial load in gravid ovaries from five females of the *Culex pipiens molestus* Celestin strain using quantitative PCR (qPCR). The average *Wolbachia* abundance, estimated by normalizing *wsp* to the host reference gene *ace2*, was 43.1 copies per host genome equivalent—indicating a high bacterial abundance in ovaries suitable for long read sequencing. qPCR also provided insights into the relative copy number of *Wolbachia*-associated mobile elements. The plasmid gene *GP11* showed an average of 1.7 copies per *Wolbachia*, suggesting nearly two plasmid copies per bacterial genome and the phage marker *GPW*, representative of the WO connector/baseplate module, exhibited an average of 9.5 copies per *Wolbachia* (S1 Table).

Fluorescent *in situ* hybridization (FISH) using *Wolbachia*-specific 16S rRNA probes was then performed on ovarian tissues from the same mosquito strain to complement molecular estimates. Observations revealed a strong and widespread bacterial signal across the ovary, with *Wolbachia* clearly visible around oocyte nuclei and within follicular cells (Fig 1).

**Fig 1:**
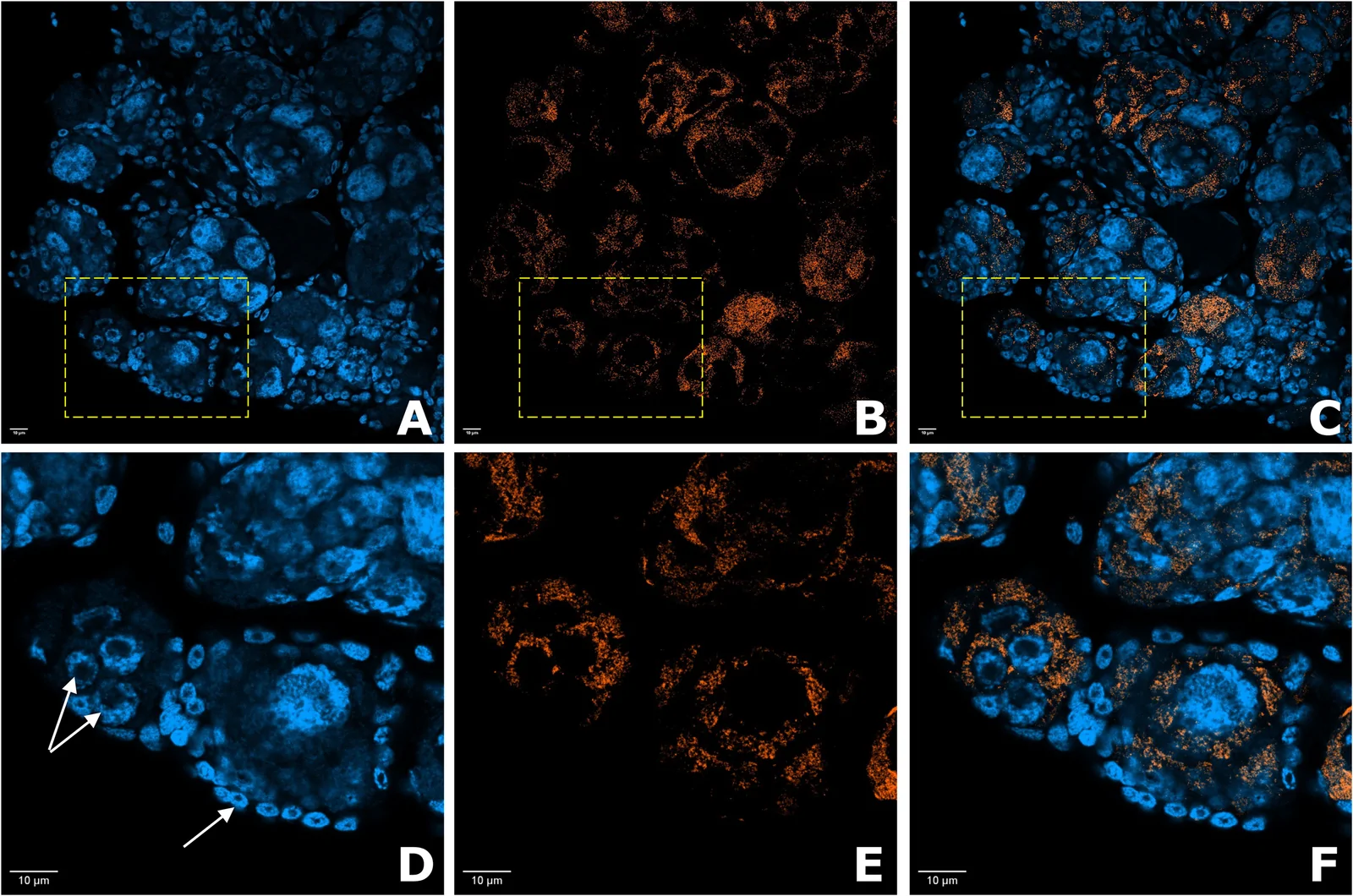
FISH *Wolbachia* visualization in the ovaries. *Wolbachia* are located in the ovarian follicles (A-F). Colored dashed boxes (A to C) indicate area of magnification (x2.6) for images below (D to F) where structures at the ovarian tip that may correspond to the germarium, nurse cells, follicle cells, and/or oocyte are noted with arrows. It is composed of oogonia that mature and increase in diameter during the ovary maturation. *Wolbachia* is imaged with an Atto 488-labeled probe targeting the *Wolbachia* 16S rRNA gene (orange, B and E), and DNA is stained with DAPI (blue, A and D). C and F are overlays.

Overall, data confirmed both the high bacterial density and the homogeneous spatial organization of intracellular *Wolbachia* in the ovaries of *Culex pipiens molestus*, supporting the relevance of this tissue for genome reconstruction.

### Nanopore sequencing of a single ovary sample for the reconstruction of *w*PipCelG genome

Nanopore sequencing of dissected ovaries from a gravid *Culex* female generated a total of 309,559 reads (median read length: 1,223 bp; maximum read length: 119,034 bp). Detailed sequencing, filtering, and read assignment statistics are provided in S2 Table. Alignment of raw sequences to the spiked in lambda phage genome showed a mean read accuracy of 94.95%, corresponding to an estimated error rate of ~5%, consistent with expectations for high quality ONT libraries [40]. The initial assembly produced a *Wolbachia* Metagenome-Assembled Genome (MAG, recruiting 24,830 reads representing 8.43% of total reads) composed of 24 contigs with 91.5% completeness and 1.4% redundancy, estimated from the bacterial single copy core genes collection of Campbell *et al*. [41], consistent with *Wolbachia* endosymbiont genomes [9,42]. Comparative pangenome analysis with three reference *w*Pip genomes confirmed high sequence similarity across aligned regions, with the newly assembled genome *w*PipCelG sharing 99.84% identity with *w*PipPel (group I of *w*Pip), 99.83% with *w*PipJHB (group I) and 99.63% with *w*PipMol (group IV). Phylogenetic analyses based on six diagnostic loci further assigned *w*PipCelG to *w*Pip group I, consistent with its close relationship to both *w*PipPel and *w*PipJHB (see S1 Fig). The resulting pangenome, comprising 1,166 gene clusters based on BLAST homology, revealed that *w*PipCelG shares 1,056 gene clusters with the three reference genomes (conserved *w*Pip core). Among accessory genes, 10 gene clusters were unique to *w*PipCelG, while 8 gene clusters present in all three references were absent, suggesting both specific acquisition and potential gene loss (S2 Fig and S3 Table).

As described in the Method Section, a manual refinement scaffolding step was applied to improve assembly quality by removing zero-coverage and redundant contigs. Read mapping and pairwise BLASTn comparisons revealed that ten small contigs either exhibited negligible coverage or showed 100% sequence identity to internal regions of larger contigs (S4 Table). These were considered likely assembly artefacts or redundant fragments and were excluded from the final genome assembly. Among the 14 remaining contigs, contig_2 (1,186,930 bp) displayed strong homology to the *Wolbachia* core genome of *w*PipPel, while contig_9 (18,485 bp) corresponded to a dimeric structure of pWCP plasmid. The remaining 12 contigs appeared to include WO prophage regions based on gene content.

### pWCP plasmid is recovered as a dimeric structure

The reconstructed plasmid of contig_9 measured 18,485 bp, approximately twice the size of the monomeric pWCP reference (9,228 bp; [9]). Its sequence was consistent with two complete pWCP units arranged consecutively in the same orientation, forming a simple head to tail tandem configuration with no additional sequence or rearrangement between the two units. The assembled sequence revealed a high degree of structural conservation with the reference (99.77% identity), with a 16-nucleotide insertion in the VNTR region corresponding to the addition of a tandem repeat (increasing from six to seven copies) (S3 Fig). A few additional differences were observed, including three insertions, one deletion, and a C to T substitution upstream of the VNTR but overall, the assembled plasmid (contig_9) was highly similar to the reference plasmid pWCP, consistent with the strong conservation previously reported [26].

Mapping of Illumina short reads from the three additional pairs of ovaries (*Culex pipiens molestus*, Celestin strain) to the *w*PipCelG assembly indicated that three single nucleotide insertion/deletion mutations observed in the tandem plasmid contig were in fact attributable to basecalling errors specific to Nanopore sequencing, particularly in the homopolymeric regions. These errors affected the accurate counting of repeated identical bases, leading to false insertions or deletions in the assembled sequence. A single base substitution (C instead of T) detected within a variable number tandem repeat (VNTR) region appeared to result from the presence of one additional repeat unit in the assembly, which likely introduced an alignment ambiguity rather than reflecting a true point mutation at this position.

### Long reads support the coexistence of monomeric and multimeric plasmid forms

To investigate long reads matching accurately to the plasmid independently of any assembly, we selected all reads mapping to contig_9 of over 9,000 bp, giving a total of 24 high-quality long reads (with a MAPQ = 60). Alignment of these long reads to the pWCP reference yielded a read accuracy estimation of 94.53%, consistent with the general error rate measured from reads mapped to the lambda genome. The 24 long reads were individually aligned to the monomeric version of pWCP to distinguish between monomeric and dimeric forms. Among them, four raw reads (>14 kb) were consistent with a dimeric or multimeric plasmid configuration, while 20 shorter reads (<11kb) were compatible with the canonical monomeric plasmid form. However, some reads could also originate from larger multimeric circular structures that were only partially sequenced and therefore not long enough to unambiguously distinguish between monomeric and multimeric configurations. We represented these four reads (Fig 2A) and the seven longest reads (Fig 2B) supporting a ca. 18kb and 9kb plasmid, respectively, in Fig 2. Notably, two long reads, read 1 and read 2 measuring 22,496 bp and 19,413 bp, respectively, covered more than twice the length of the monomeric plasmid, providing read-level support for the tandem organization observed in the assembly (Fig 2A). In addition, read 3 (18,092 bp) aligned contiguously to the reference sequence for 14,123 bp but the remaining region matched pWCP with gaps (not highlighted on Fig 2A).

**Fig 2:**
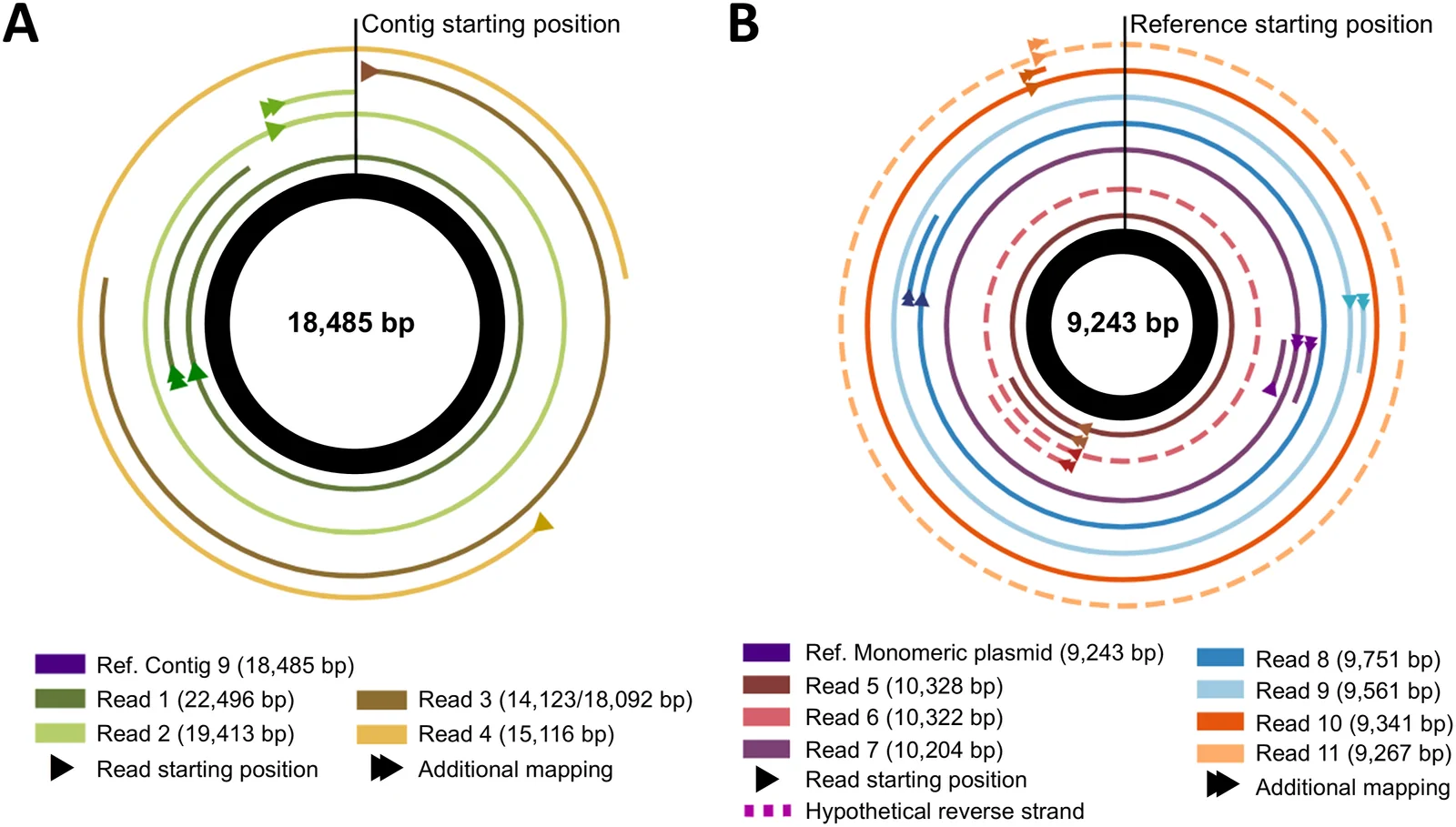
Identification of monomeric and dimeric pWCP forms in *w*PipCelG through long read mapping. (A) Long reads longer than 14,000 bp mapped to the 18,485 bp dimeric pWCP contig. Reads aligned contiguously over their full length against the reference sequence except read 3 which mapped contiguously to the reference for 14,123 bp but the remaining region matched pWCP with several gaps (not highlighted). (B) Long reads longer than 9,000 bp mapped to the 9,243 bp monomeric form of pWCP of *w*PipCelG (half of Contig 9). In both panels, triangles indicate the starting position of each read, as retrieved by alignments to the references, while double triangles mark additional alignments from the same read. Read paths should be interpreted from the innermost circular layer to the outermost circular layer. For each layer, reading begins at the (simple or double) triangle arrow marker, and proceeds either in the sense or antisense direction as indicated by the arrow orientation. Presence of a full circle in the circular layer indicates a complete traversal of the monomeric or dimeric plasmid reference sequence, ending at the starting arrow position, before continuing to the next, outer layer.

### The observed plasmid structures may be associated with plasmid replication

We examined the start and end positions of the 24 plasmid reads and found that most started and ended at apparently random positions along the plasmid (S5 Table). However, two long reads of nearly identical length but opposite orientation (Read 5: 10,328 bp and read 6: 10,322 bp) both started and ended at the same positions (Fig 2B). This observation could suggest that these reads originated from the forward and reverse strands of a single DNA molecule undergoing replication while adopting a coiled conformation. The presence of overlapping regions within these reads also aligns with a double-stranded plasmid possibly replicating via a theta-like mechanism. A scheme for how these sequences may arise is provided in S4B Fig. In addition, another long read (Read 7: 10,204 bp) exhibited a complex structure, beginning with a ~ 400 bp region in antisense orientation, followed by the same sequence in sense orientation, then continuing through the rest of the plasmid and ending with an additional copy of the initial region in sense. This arrangement also supports the possibility of replication associated structural intermediates, and a schematic hypothesis of such a configuration is presented in S4C Fig.

Independently, we analyzed the reference pWCP sequence using OriV-Finder [43] to further explore potential replication features that could explain such multimeric configurations. This analysis identified a type II origin of replication (oriV) located between positions 6,363 and 6,513 bp (S5 Fig). This region overlapped with the previously annotated VNTR composed of 16-bp tandem repeats and displayed features consistent with iteron-based replication initiation, a mechanism previously described in association with multimeric plasmid configurations.

Together, the multimeric organization of pWCP in the assembly, the unusual structures observed in several long reads, and the predicted oriV is consistent with potential replication-associated plasmid structures.

### Wolbachia and WO genomes exhibit complex structural organization

The *Wolbachia* core genome reconstructed from contig_2 (1,186,930 bp) exhibited an average coverage of approximately 30X. The bacterial origin of replication was identified between positions 426,616 and 427,120 bp in this contig. In addition to this single large contig, phage-rich regions were recovered as multiple smaller contigs (12 contigs), reflecting the challenge of assembling highly repetitive prophage sequences (Fig 3A). This observed fragmentation and incomplete recovery of *Wolbachia* genomes despite the successful long-read sequencing of a single individual without prior amplification highlighted the structural complexity of phage loci. More specifically, the fragmented prophage regions appear to occur distantly from the predicted bacterial origin of replication (*oriC*) [44]. Consistent with this pattern, phylogenetic analysis of the large serine recombinase places the *w*PipCelG WO element within the sr3WO clade as defined by Bordenstein & Bordenstein (2022) [12] (S6 Fig), a group for which distal phage integration relative to *oriC* has been reported. Within contig_2, phage-related genes were detected at both ends of the contig (Fig 3A). Among them, a *cifB* gene from phylogenetic group Type IX [23] was identified at positions 6,131–8,827 (corresponding to WP0462 in *w*PipPel), along with several other phage-associated genes, including two head structure genes located at positions 1,153,567–1,155,632 (S6 Table). Additionally, a partial sequence of an *Octomom-*like region was identified towards the terminal end of contig_2 (from 1,167,601–1,186,910 bp). This fragment contained ‘toxicity-associated ‘domains, predicted eukaryotic-like toxin, including WP1346 and WP1348 together with a partial WP1349 from *w*PipPel interspersed with transposase sequences (WP1345 and WP1347). This genomic organization is similar to what has been described for the *Octomom*-like region in *w*PipPel [12]. In addition, several long reads mapping to the terminal phage-associated region of contig_2 also partially mapped to smaller WO-rich contigs, notably contig_10 and contig_22. However, closer inspection of these reads revealed that they were relatively short (mostly 5–6kb) and that their WO-associated portions aligned to multiple phage rich contigs, often at different positions along the same contig, due to the highly repetitive nature of these regions. These ambiguous alignments prevented the reads from being unambiguously assigned to a specific WO region or genomic arrangement. Thus, although these reads show sequence similarity between the terminal phage-associated region of contig_2 and other WO-rich contigs, they do not allow us to resolve their precise structural organization or integration state.

**Fig 3:**
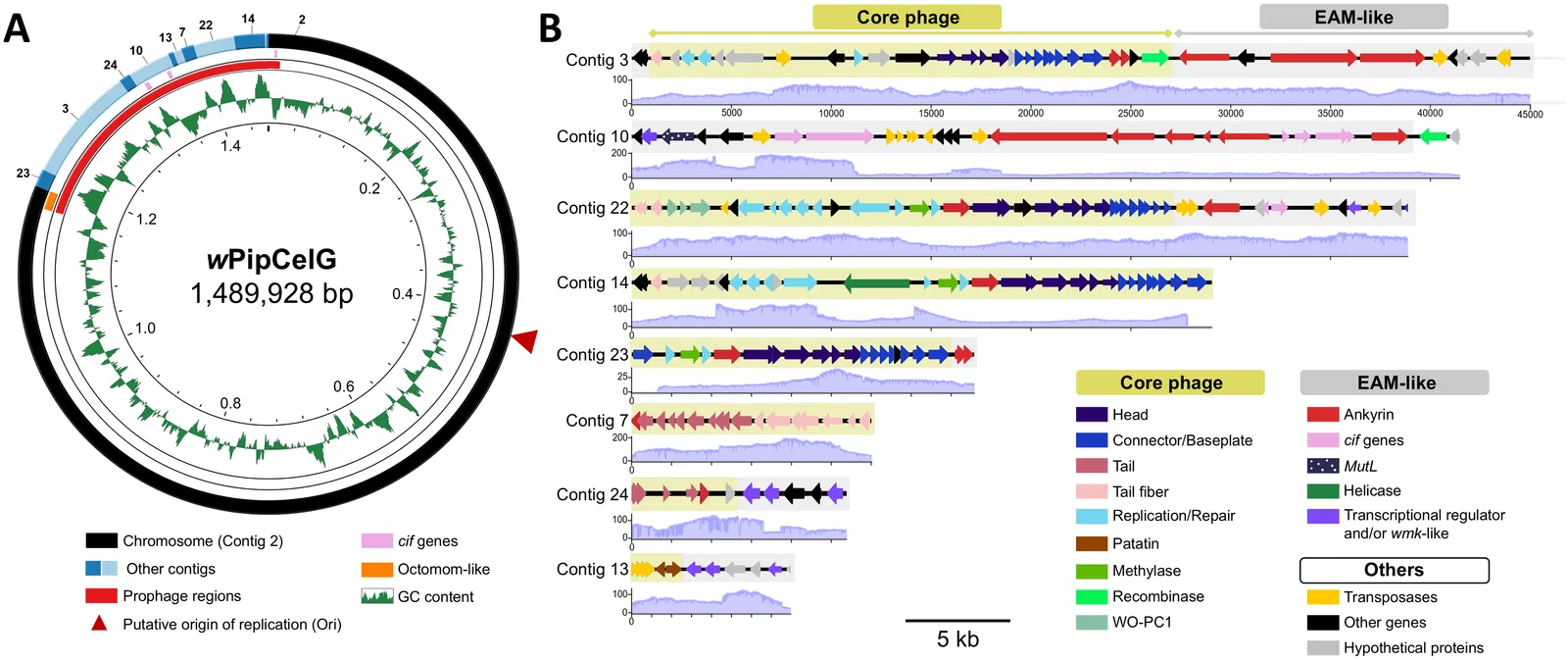
Genome organization and annotation of phage-associated contigs in *w*PipCelG. (A) Schematic representation of the *Wolbachia* MAG *w*PipCelG, showing the position of the predicted origin of replication (Ori) and the fragmented contigs corresponding to phage-associated regions, illustrating the overall genome organization obtained from long-read sequencing. Because the WO-associated contigs remain fragmented and their chromosomal locations could not be confidently resolved, their relative order and positioning in the circular representation are arbitrary. (B) Phage-associated contigs larger than 7,000 bp are displayed, showing their gene synteny and the sequencing coverage profile for each contig (per number of raw reads), highlighting structural organization and depth of mapping across these phage regions.

### WO phage regions exhibit extensive structural rearrangements

Following manual curation, prophage WO-associated regions identified within contig_2, together with the 12 additional contigs containing prophage WO related regions, were further analyzed, and the gene synteny, functional annotation, and sequencing coverage of the seven longest WO-rich contigs (from 7,964–11,2962 bp) are illustrated in Fig 3B. We observed core phage genes flanking EAM regions in almost all contigs, in agreement with *w*PipPel reference genome [12,35]. Most contigs exhibited non-uniform coverage, most likely due to repeated genes recruiting reads in a non-specific manner. This confirms that for a large fraction of WO regions, the assembly is unreliable and highlights the intrinsic difficulty of assembling these repeat-rich loci. A minority of contigs (e.g., contig 3, contig 22) showed near-uniform coverage.

We next focused on the relative abundance of representative genes from the main structural modules of WO phage, namely, the head, connector/baseplate, tail, and tail fiber components. For each category, we calculated the average sequencing coverage (Fig 4A), which served as a proxy for estimating copy number (Fig 4B). Head genes showed coverage levels consistent with approximately 6–7 copies, connector/baseplate genes with 6–7 copies, and tail and tail fiber genes with 3–5 copies (Fig 4B). These values suggest that the genome contains multiple intact and relic prophage elements, the latter of which have experienced gene loss and/or rearrangements in one *Wolbachia*. The higher abundance of head and baseplate genes relative to the rest of the phage WO genome is a common feature of prophage WO [45], and the intact prophage variants are compatible with putative ongoing constitutive phage activity. In addition to structural components, we also assessed the copy number of phage-associated genes involved in host manipulation and proliferation, including *cifA-cifB* and the *Octomom*-like region. On average, we estimated four copies of the Type I *cif* genes per *Wolbachia* genome, with both genes located on contig_10. The Type IV *cif* genes were detected as single copies, also located on contig_10. We counted a single copy of the *Octomom*-like region identified at the terminal end of contig_2, in agreement with assembly data. The coverage analysis also included transposase-associated genes, with 11 gene clusters identified and substantial variation in their estimated abundance. Notably, IS982 was the most highly represented, with 56 copies predicted from read coverage. Details of the read coverage and the estimated copy number of each WO phage gene are provided in S7 Fig.

**Fig 4:**
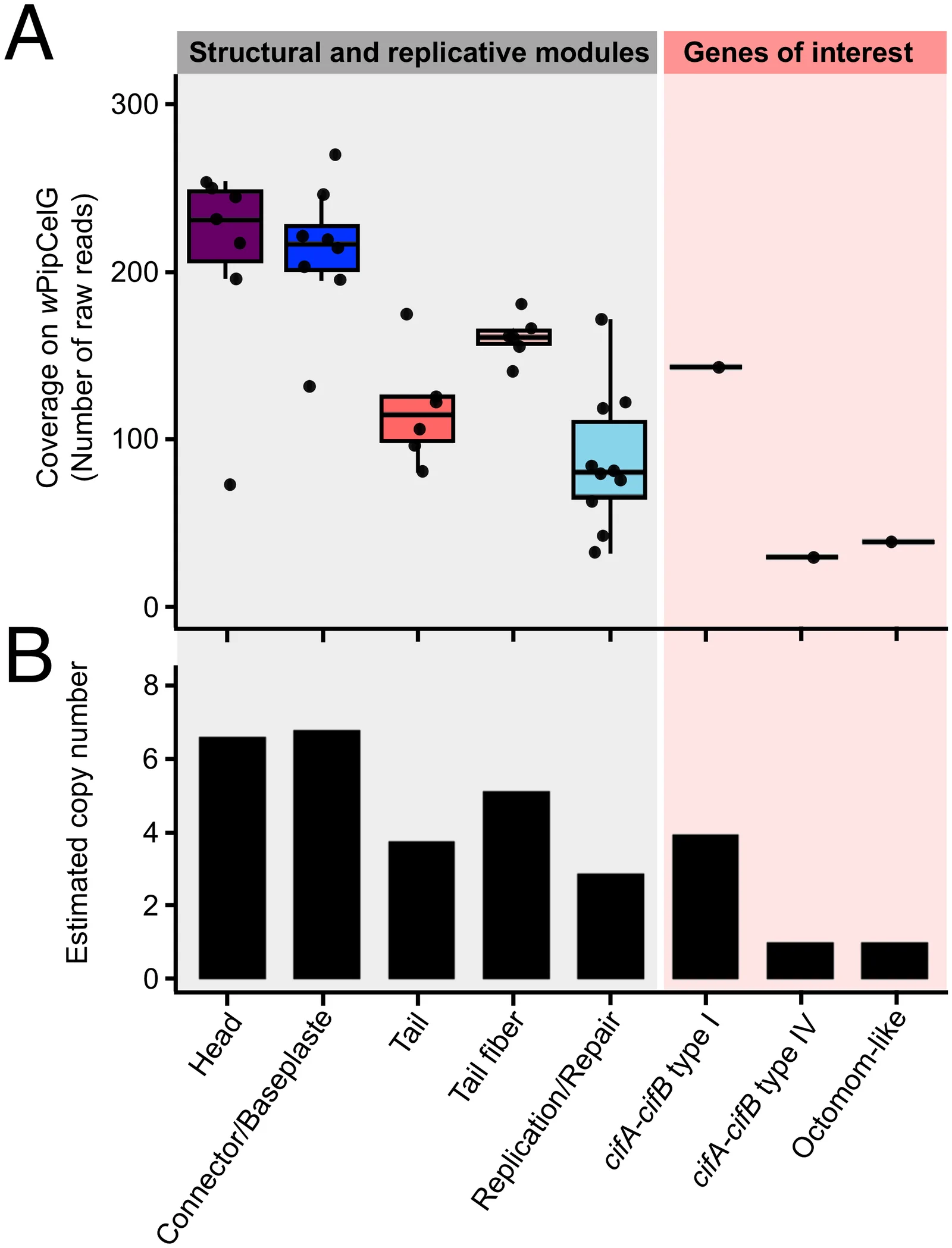
Coverage and estimated copy number of *w*PipCelG phage structural genes and particular genes of interest. (A) Filtered long reads were mapped to representative sequences of each *w*PipCelG gene cluster (defined as the longest gene sequence within the cluster from the pangenomic analysis) for “structural and replicative modules” while on unique sequences for the “genes of interest”. Coverage depth is displayed as boxplots (number of raw reads).(B) Estimated copy numbers (indicated below each boxplot) were inferred by linear regression, based on the relationship between assembled copy numbers in gene cluster and coverage in *w*PipCelG. For phage structural and replicative modules, the value shown represents the average estimated number of gene copies per module.

To further investigate these prophage-rich regions independently of any assembly, we examined gene syntenies directly from annotated raw long reads (≥10 kb). First, we noted several independent long reads (6 reads > 10kb) carrying annotations from regions assigned to WOPip1 and WOPip5 in the *w*PipPel reference on the same molecule, supporting that WOPip1 and WOPip5 are likely adjacent and/or rearranged in *w*PipCelG (Fig 5A). By contrast, these prophages are non-contiguous in *w*PipPel [12]. Within the same region, we also noted a transposase (WP1350) that is absent from the *w*PipPel reference, highlighting mobility and/or local amplification of transposases within *Wolbachia*.

**Fig 5:**
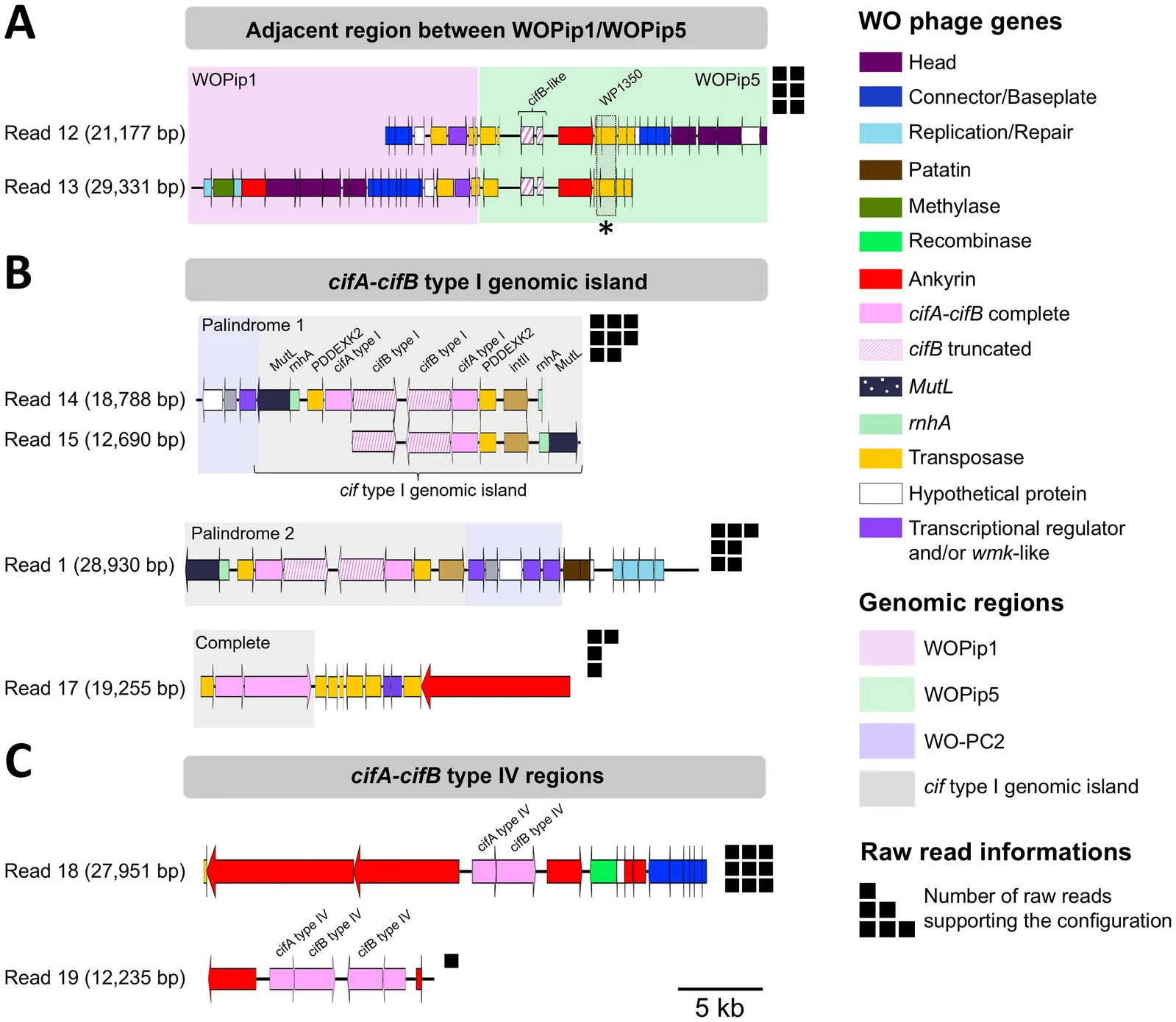
Nanopore raw-read synteny in WO phage regions: WOPip1–WOPip5 junction, *cif* type I genomic islands and *cif* type IV. (A) Read-level observations showing WOPip1–WOPip5 adjacency in *w*PipCelG. The asterisk (*) denotes a locus (transposase WP1350) that is absent in the *w*PipPel reference genome but present in *w*PipCelG. (B) *cif* genomic island configurations around *cifA*/*cifB* type I. Three distinct arrangements resolved from annotated ≥10 kb Nanopore reads: two type I *cifA/B* palindromes with truncated *cifB* and one complete *cifA–cifB* tandem identical to *w*PipPel. (C) *cif* type IV configurations. Two distinct arrangements are present from annotated ≥10 kb Nanopore reads. For each configuration (A, B, and C), one or two representative long reads were used to illustrate gene synteny. The total number of supporting reads is indicated next to each schematic by black squares. Genes are shown as oriented boxes, with colors indicating their module or functional class.

Second, we identified three configurations of the Type I *cifA-cifB* region (Fig 5B): two *cifA-cifB* palindromes with truncated *cifB* (supported by 8 and 7 reads (>10kb), respectively) and one full-length *cifA-cifB* tandem (supported by 4 > 10kb reads). The two Type I palindromic configurations also differed in their flanking genomic regions. While both shared a *mutL-rnhA-*PPDEXK2 region on one side, the opposite side differed, with PPDEXK2 - *intII* - *rnhA* adjacent to the *cif* region in Palindrome 1 and a PPDEXK2- *intII* region followed by WO-PC2 associated region in Palindrome 2. These distinct genomic contexts highlight the variable organization of the regions surrounding the Type I *cif* genes. In total, these represent five instances of the *cifA–cifB* pair. We then observed two configurations for *cif*A-*cif*B Type IV (Fig 5C), including the classical tandem configuration present in *w*PipPel (supported by eight reads) and a palindrome *cifA–cifB-cifB-cifA* supported by a single ≥10 kb read. These findings suggest rapid evolution of these host manipulation genes.

Separately, we attempted generating a “*cif* synteny long read gene-adjacency graph” (S8 Fig). Starting from the *cifA* Type I gene (WP0282 in *w*PipPel), we enumerated all observed transitions to the next gene along individual reads and annotated these transitions in each read. This analysis revealed multiple gene-order configurations emanating from *cifA*, in agreement with the non-uniform coverage and contig fragmentation observed in WO regions.

## Discussion

We performed long read sequencing on gravid ovaries from a single female mosquito individual, without prior DNA amplification, providing an optimized and unique experimental framework to explore the genomic architecture of *Wolbachia* and its mobilome. This strategy limited amplification biases, avoided the mixing of potential variants across individuals or tissues [42], and enabled the direct capture of genomic elements that are typically difficult to access, including rare or less frequent forms of the mobilome. Additional scrutiny of both raw long read and short read datasets, obtained from independent samples, allowed us to curate the assembly and highlight a set of active MGE in the *Wolbachia* endosymbiont from *Culex*. First, both monomeric and candidate dimeric forms of pWCP were identified, consistent with pWCP being a putative self-replicative element within a highly dynamic mobilome, although the available data could not exclude possible recombination or technical sequencing artifacts. Second, WO-associated regions appeared as substantially more diverse and structurally complex than previously represented in available *w*Pip reference genomes. In line with this, the coexistence of multiple structural variants within a single individual suggests that currently available assemblies may underestimate the extent of intra-host genomic heterogeneity in *Wolbachia* populations. However, despite these methodological improvements, several highly repetitive WO-associated regions remained only partially resolved, illustrating the persistent technical challenges associated with reconstructing complex mobilome architectures even using optimized long-read sequencing approaches.

### Plasmid structure and possible replication

The assembly of a putative dimeric plasmid, supported by several long reads spanning two or more copies of the plasmid, suggests that this element may naturally exist in multimeric and monomers forms, including dimers, within the cell. However, the biological origin of these multimeric configurations remains uncertain. Several non-exclusive hypotheses can be proposed to explain this observation.

On one hand, these multimeric forms may result from plasmids undergoing replication at the time of DNA extraction. In this context, specific replication dynamics might occur at this particular developmental stage (gravid ovaries), where increased replication activity favors the capture of intermediate forms during sequencing. Notably, both major plasmid replication mechanisms, theta-replication and sigma-replication, are known to generate concatemers, especially when termination of replication fails [46–48]. In line with this hypothesis, sequence analysis of the plasmid revealed a putative origin of replication (oriV) located within a region previously annotated as a Variable Number Tandem Repeat (VNTR), composed of 16-nucleotide tandem repeats. The exact position of this origin within the repeated region, along with the conservation and structure of the motifs, is consistent with a replication control mechanism involving iterons, as described in theta-replicating plasmids [49]. Iterons are short repeated sequences that serve as binding sites for replication initiator proteins, facilitating local DNA unwinding and regulating plasmid copy number [49]. Although no *rep* gene was identified within the plasmid itself, the bacterial host encodes a *repA* prophage gene, which could potentially participate in initiating replication. In addition, the presence of a *parA-like* gene upstream of this VNTR region suggests an active partitioning system, which is consistent with the behavior of low-copy-number plasmids [50]. Taken together, this genomic context, along with the detection of long reads slightly larger than the monomeric plasmid and exhibiting overlapping region, is compatible with the hypothesis of a theta-type replication mode, which has not been previously proposed for this plasmid.

On the other hand, these forms may originate from unresolved recombination events [51,52]. In many low-copy, theta-replicating plasmids, specific resolution systems (such as *res* or *cer* sites) are responsible for efficient multimer separation [51,53]. Although no such site was detected in the analyzed sequence, their apparent absence does not exclude the possibility of a non-canonical resolution mechanism. Without such a system, persistent multimeric forms could impair proper plasmid segregation during cell division and represent a burden for the host cell [54]. Nevertheless, some studies suggest that these forms may not necessarily destabilize the plasmid but could impose a significant energetic cost due to unregulated amplification [55].

Finally, we note that a technical origin of the multimeric pWCP sequences cannot be formally excluded. We considered three main types of artifacts described for Nanopore sequencing: ligation-derived, -*in silico* chimeras, and foldback artifacts [56,57]. Here, no internal adapter was detected in the supporting reads, the pWCP units occurred consecutively in the same orientation across several independent reads, and no inverted organization characteristic of foldback artifacts was observed. An *in silico* chimera would require two independently sequenced pWCP molecules to pass through the same nanopore in rapid succession and to be concatenated at compatible sequence coordinates, reproducing the uninterrupted head-to-tail tandem organization observed here. This combination of events appears unlikely given the variable endpoints of the pWCP reads and the relatively low representation of pWCP sequences in our metagenomic dataset. Together with the recovery of the same tandem organization across multiple assembly configurations, these observations make these known technical artifacts less consistent with our data, although an uncharacterized sequencing artifact cannot be fully excluded.

### Assembly limitations and underestimation of WO phage diversity

Our reconstruction and analysis of a novel *Wolbachia* genome *w*PipCelG from *Culex pipiens molestus* at the organ level highlighted persistent challenges in assembling prophage regions, even under optimized experimental conditions involving long read sequencing from a single reproductive organ without prior DNA amplification. Twelve contigs containing phage genes were reconstructed, reflecting the high fragmentation of this genome. This fragmentation was also consistently observed across the seven additional assemblies generated using alternative read subsets and assembly parameters. This likely resulted from the highly repetitive nature of prophage regions and possibly the presence of active phage particles [12,35]. Coverage profiling across WO regions further revealed a discrepancy between the expected copy number and the number of WO segments recovered by assembly, consistent with the underrepresentation of prophage diversity and/or free phage WO at a constitutive level. This pattern accords with prior observations [32] of mismatches between *cif* copy numbers estimated by qPCR and those annotated in the *w*PipPel reference, indicating that assemblies alone can be incomplete for multicopy loci. However, despite these observations, the current dataset does not provide sufficient structural resolution to confidently discriminate between multiple integrated tandem prophages, episomal WO forms or potentially active lytic phage particles.

In our dataset, the overall genomic structure of *w*PipCelG and *w*PipPel (both *w*Pip-I) is broadly similar, yet coverage in *w*PipCelG is consistent with approximately four copies of the Type I *cifA-cifB* pair (consistent with the 4–6 range reported for *Culex pipiens* [32]), despite single-copy annotations in both assembled genomes. Taken together, these observations illustrate how copy number estimation based solely on genome assemblies may misrepresent the true genomic landscape, particularly for multicopy genes embedded within repetitive regions. In line with this, our read-level inspection revealed multiple alternative syntenies around *cif* loci (Types I and IV), and the number of distinct arrangements does not fully track the coverage-based copy number estimates.

Recent long read studies across *w*Pip groups have likewise reported distinct syntenies around *cif* genes [58]. In the *w*Pip-I (Tunis strain), three different *cif* adjacent environments were documented and termed *cif*-Type I genomic islands. Consistent with these findings, inspection of the raw long reads from *w*PipCelG (*w*Pip-I) also identified distinct *cif*-Type I genomic islands. While the overall pattern agrees with the *w*Pip-I (Tunis) report [58], the exact compositions are not identical: one additional configuration observed in *w*PipCelG (Palindrome 2) was not previously reported, whereas one of the previous configurations (Tunisp-d) is absent from our dataset, suggesting that variability in these regions is likely not dependent on *w*Pip group.

Notably, the number of distinct *cif* configurations identified using long reads exceeds coverage-based copy number estimates (five Type I *cifA-cifB* tandem vs. four copies inferred from coverage; for Type IV *cif*, coverage supports one copy yet two configurations were observed). These modest mismatches between coverage-based copy number and the number of *cif* configurations could suggest the presence of distinct syntenic configurations coexisting among *Wolbachia* cells (i.e., subpopulations) within the same host. Overall, these observations point to structural heterogeneity both between mosquito strains within the same *w*Pip group and within individual hosts, where distinct *Wolbachia* genomes bearing different prophage organizations may co-occur and/or lytic WO forms may contribute additional configurations. We note that some configurations are supported by few long reads, and although chimeric molecules cannot be excluded, such patterns may reflect rare states present at low frequency. Given the low sequencing depth of this sample, with configurations supported by at most eight reads (>10kb), single occurrence observations remain noteworthy and potentially meaningful, warranting further investigation. The fast evolving long read sequencing technologies (*e.g.,* R10.4.1 flow cells and Dorado basecalling) may allow much deeper sequencing of such low biomass samples in a near future, shedding light on these and other rare phage WO variants. Nanopore adaptive sampling might also be considered for targeted enrichment of low-abundance phage configurations. However, the extremely large size of the *Culex* genome compared with *Wolbachia* genomes—a roughly 400-fold difference—could potentially result in substantial consumption of sequencing capacity when targeting such disproportionately large host genomes, which might limit the recovery of low-abundance sequences. Nevertheless, the feasibility and potential of this approach would warrant experimental evaluation.

Based on these data, it is conceivable that a substantial portion of WO phage diversity and structure remains undetected by current assembly approaches, even when using high-quality long read data. In support of this idea, our qPCR results indicate in addition to a high *Wolbachia* load in the gravid ovaries of Celestin strain, with an estimated 40 bacteria per host cell, an average of approximately 9 copies of a targeted phage gene (present in 3–4 copies in the *w*PipCelG and *w*PipPel assemblies, respectively). These values are consistent with the coverage-based estimations afforded in this study and further the idea that phage diversity and/or abundance in this strain and possibly other *Culex* ones is strongly underestimated.

Such underappreciated WO diversity may have important implications for the biology and evolution of *Wolbachia* in *Culex* mosquitoes. For example, because *cif* genes are embedded within WO-associated regions, structural variability across prophage loci could contribute to the diversification of CI-associated regions and potentially participate to the diversity of incompatibility patterns observed among *Culex pipiens* populations [58]. More broadly, prophage-associated regions are increasingly recognized as important contributors to genome evolution in *Wolbachia*, notably through recombination and horizontal transfer processes [12,58]. In line with this, comparative analyses across *Wolbachia* have shown that larger genomes are often associated with increased prophage content and facultative symbiotic lifestyles [8], supporting the idea that WO-associated regions may contribute substantially to genomic diversification and host interaction evolution.

## Conclusion

This study illustrates the importance of critically assessing assembly outputs, especially when working with highly repeated and mobile regions prone to structural complexity within an endosymbiont genome. When feasible, the combination of long read and short read data, along with direct inspection of raw reads and coverage profiles, offers a valuable strategy to investigate biological genomic features in an assembly-independent manner. In our case, long read sequencing without prior DNA amplification enabled direct access to single-organism genomic information, while complementary short read sequencing from additional samples helped refine and validate the plasmid assembly. Thanks to this approach, we provide here a new genome assembly for *Wolbachia* of *Culex pipiens molestus*, characterized both at the genomic level (with some WO-rich regions still partly unresolved) and through *in situ* visualization within the host tissue. In addition, our observations further illustrate the dynamic nature of the *Wolbachia* genome in *Culex pipiens*, particularly regarding its mobilome. The structural variability observed across both plasmid and phage elements support that these components may play an ongoing and significant role in the genomic plasticity of this symbiont. Notably, the pWCP element, is here suggested as a putative replicative plasmid, carrying a functional origin of replication and occurring in both monomeric and dimeric forms, although we remain cautious about this interpretation, as the potential occurrence of uncharacterized artifacts cannot be fully excluded. These findings echo previous suggestions that, despite the streamlined nature typically associated with intracellular genomes, the acquisition and activity of MGE may serve as a source of diversification and adaptive potential for *Wolbachia*, helping to partially counteract the reductive pressures of its intracellular lifestyle [7,59].

## Materials and methods

### Quantification and visualization of *Wolbachia* prior to sequencing

We assessed the abundance and localization of *w*Pip in ovaries from *Culex pipiens molestus* (Celestin strain) to ensure the tissue suitability for long read sequencing. This mosquito strain was collected in Montpellier (France) in 2023 and has since been maintained as a laboratory colony under standard insectary conditions [27]. These exploratory analyses were conducted prior to DNA extraction for Nanopore sequencing.

Gravid ovaries from *Culex pipiens molestus* Celestin individuals were dissected following previous methods [60], and total DNA was extracted using the DNeasy Blood and Tissue Kit (Qiagen, Hilden, Germany), according to the manufacturer’s instruction. Quantitative PCR (qPCR) was performed to assess the abundance of *Wolbachia* and associated MGE, targeting four genes: *wsp* (*Wolbachia* surface protein), *GP11* (plasmid pWCP), *GPW* (a gene associated with the WO phage connector/ baseplate module), and *ace2* (mosquito acetylcholinesterase gene, used as host reference). Among these, *wsp*, *GP11*, and *ace2* are present as single-copy genes in their respective genomes, while *GPW* occurs in multiple copies (four in the *w*PipPel reference genome). All qPCR conditions (thermal cycling, reagents, instrumentation, and quantification based on synthetic plasmid standards) followed the protocol described previously [27]. The relative abundance of *Wolbachia* was calculated by normalizing *wsp* to the host gene *ace2*, as in the referenced study. To estimate the relative abundance of MGEs within the bacterium, *GP11* and *GPW* copy numbers were normalized to *wsp*.

Fluorescent *in situ* hybridization (FISH) was performed on dissected ovaries in 1X phosphate-buffered saline (PBS), following the same dissection protocol. Samples were fixed in 4% paraformaldehyde (PFA) for one to three hours at 4 °C, then washed two to three times with 1X PBS to remove residual PFA. Following washes, samples were stored at −20 °C in 1X PBS containing 50% ethanol until hybridization. Hybridization was carried out overnight at 46 °C in 180 μL of hybridization buffer (35% formamide, 0.9 M NaCl, 20 mM Tris-HCl [pH 7.6], 0.01% SDS) supplemented with 20 μL of each probe at 30 ng/μL. Two *Wolbachia*-specific 16S rRNA probes were used: W2: 5′-CTTCTGTGAGTACCGTCATTATC-3′ and W3: 5′-AACCGACCCTATCCCTTCGAATA-3′, both labeled with Atto 488 (Eurofins). After hybridization, samples were washed twice in 1 mL of pre-warmed washing buffer (20 mM Tris-HCl [pH 7.6], 70 mM NaCl, 5 mM EDTA, 0.01% SDS) for 30 min and 15 min respectively at 48 °C, followed by an additional 15 min wash in 1X PBS at room temperature. Samples were stained with DAPI (1 μg/mL, Sigma-Aldrich), rinsed in 1X PBS, dried, and mounted using ProLong Gold Antifade Reagent (Invitrogen, ThermoFisher Scientific). Imaging was performed using a Leica Stellaris confocal microscope equipped with a 63x objective.

### Long read sequencing

Gravid ovaries from a single *Culex pipiens molestus* (Celestin, strain) were dissected and genomic DNA was extracted using a phenol-chloroform protocol optimized to preserve high molecular weight fragments. The use of a single ovary was intended to characterize an individual *Wolbachia* population while avoiding pooling potentially distinct variants from multiple mosquitoes, which may be particularly relevant given previous observations of SNPs within core *Wolbachia* genes among independent *Culex pipiens* ovary samples [42]. DNA concentration was measured with a Qubit fluorometer (High sensitivity DNA kit, Thermo Fisher Scientific) and the fragment size distribution was assessed with a Bioanalyzer (Agilent Technologies). Extracted DNA was stored at -20°C until sequencing. Library preparation for Oxford Nanopore sequencing was carried out using the Ligation Sequencing Kit for genomic DNA (SQK-LSK110, Oxford Nanopore Technologies), following the manufacturer’s protocol. PCR amplification is often used to increase DNA yield when working with low-biomass samples but may introduce amplification biases and alter the relative representation of genomic sequence [61–63]. To minimize these potential artefacts, a direct long read sequencing strategy was employed without prior whole genome amplification. Because the amount of ovarian DNA recovered from a single mosquito was below the recommended input for Nanopore library preparation (1µg genomic DNA), *lambda* phage DNA was added as carrier DNA to reach the minimum required DNA quantity (detailed in S7 Table). Similar carrier DNA approaches have previously been proposed for ultra-low input sequencing applications to improve library preparation efficiency while preserving amplification free workflows [9,62]. Although carrier DNA may reduce the proportion of informative reads originating from the biological sample, it can subsequently be computationally removed from downstream analyses. A blast search between the lambda sequence (NCBI: J02459.1) and reference *w*PipPel genome (GCF_000073005.1) to look for potential similar similarity revealed no homology, ensuring that downstream analyses remained unaffected. The sequencing run was performed on a MinION Mk1C using a FLO-MIN106 flow cell and lasted 24 hours.

### Nanopore data processing and *Wolbachia* assembly

Raw Nanopore sequencing data was first base-called using Guppy v6.5.7 (Oxford Nanopore Technologies) with the Super Accuracy (SUP) model to convert FAST5 files into FASTQ format while simultaneously trimming residual sequencing adapters. A preliminary quality control step was then performed using NanoPlot [64] to assess the distribution of read quality scores and lengths. Reads mapping to the *Culex pipiens molestus* reference genome (NCBI: CM044968.1) and the lambda phage genome (NCBI: J02459.1) were identified using Minimap2 [65] and removed with Illumina-utils [66]. To prevent the potential removal of *wPip* sequences due to close identity with the host, we masked parts of the *Culex pipiens molestus* genome with over 0.85 identity to *Wolbachia pipientis* over 80 bp length, or with entropy lower than 0.7, using the BBtools [67] suite. This filtering step aimed to focus downstream bioinformatic analyses specifically on *Wolbachia* derived reads, thereby optimizing computational efficiency.

The resulting filtered long reads were assembled using Flye 2.9.1 [68]. The assembly used for subsequent analyses was generated using the --meta and --nano-raw parameters and is fully documented in S1 Notebook. To assess assembly robustness, seven additional assemblies were generated using either the complete filtered read dataset or reads >2,000 bp (four different configurations), with different combinations of --nano-raw, --nano-hq --read-error 0.05, --meta and --keep-haplotypes options. Overall, these additional assemblies supported the main findings obtained with the assembly presented here and used for subsequent analyses, including the tandem organization of pWCP, the main chromosomal contig similar in length (~1.19–1.20 Mb), and the persistent fragmentation of WO-associated regions. Detailed parameters and assembly comparisons are provided in S2 Notebook.

Assembled contigs were then imported into anvi’o v8 [69] for genome annotation and interactive visualization. Functional annotations were performed using the KOfam (KEGG Orthology) [70] and COG (Clusters of Orthologous Groups) [71] databases. A taxonomic classification of predicted genes was carried out with Centrifuge [72], in order to verify the identity of contigs and guide genome binning.

We manually refined the resulting *Wolbachia* MAG that we hereafter named *w*PipCelG (for *Wolbachia* in *Culex pipiens molestus*, Celestin strain, gravid ovaries), using the interactive program “anvi-refine”, thereby generating a novel reference genome for the *Culex pipiens* complex (Celestin strain, gravid female). Lastly, we applied Proovframe [73] to correct fragmented open reading frames (ORFs) resulting from long read sequencing, with the goal of improving the global quality of the genome annotation. These analyses are reported in S1 Notebook.

### Pangenomic analyses

We conducted a pangenomic analysis using anvi’o v8 following the established workflow detailed in https://merenlab.org/2016/11/08/pangenomics-v2/. The analysis included three reference *w*Pip genomes: *w*PipPel (GCF_000073005.1), *w*PipJHB (GCF_000156735.1), and *w*PipMol (GCF_000723225.2). The pangenome was constructed using the anvi-pan-genome command with the parameters --use-ncbi-blast to compute pairwise sequence similarity using BLASTp, with default sequence similarity threshold –min-bit 0.5, and with --mcl-inflation 10 to control the granularity of gene clustering. This high inflation value was chosen to ensure stringent clustering, so that only highly similar gene sequences were grouped into the same gene cluster (defined as groups of homologous genes based on amino acid sequence similarity). We also computed pairwise average nucleotide identity (ANI) and sequence coverage between *w*PipCelG and each reference genome using the pyANI toolkit [74] with the ANIm symmetric method [75] to complement the gene-based comparison.

The resulting pangenome comprised 1,166 gene clusters, among which 114 clusters from *w*PipCelG were annotated with functions matching previously characterized WO phage genes (WOPip1 to WOPip5 [12],). For each of these phage-associated clusters, all paralogous sequences from *w*PipCelG were extracted. The longest sequence among each set of paralogues was selected as the representative gene for downstream analysis.

We focused on genes belonging to key WO structural and replicative modules—*head*, *connector/baseplate*, *tail*, *tail fiber* and *replication/repair* —as well as on genes involved in reproductive manipulation and proliferation, such as *cif* and *Octomom* genes, respectively, to estimate the number of prophage gene copies [12]. Filtered long reads were mapped to the representative sequences of each gene cluster (defined as the longest gene sequence within the cluster from the pangenomic analysis) for structural and replicative modules while on unique sequences for *cif* and *Octomom* genes, using Minimap2 [65], and the coverage for each gene was calculated. Given the difficulty to assemble repeated regions and to convert coverage into estimated copy number, we performed a linear regression analysis using the read coverage of *w*PipCelG genes as predictors and their actual copy number within the genome as the response variable. The resulting model yielded an R² of 0.707, a slope of 0.03, and an intercept of –1.09. This regression equation was then used to estimate the copy number of each WO phage gene cluster based on its coverage. All data processing steps, model training, and coverage calculations are provided in S3 Notebook.

### Refinement and annotation of contigs

The initial *w*PipCelG genome assembly performed using Flye 2.9.1 [68] produced an initial set of 24 contigs after which we conducted a manual refinement step. Raw reads were aligned back to the assembled contigs using Minimap2 [65] with default parameters for Oxford Nanopore reads. Read alignments were then filtered with Samtools v1.10 [76], keeping only reads with a mapping quality of at least 2 (MAPQ ≥ 2), in order to exclude low confidence and secondary alignments. We assessed the effect of this procedure in S4 Notebook. For each contig, summary statistics including depth of coverage, number of mapped reads, read length distribution, and N50 were calculated (see S4 Table). Contigs with no or negligible coverage were flagged as potential artefacts. To further assess redundancy, a pairwise BLASTn analysis was performed between all contigs. Contigs showing 100% identity to internal regions of larger contigs were considered redundant or misassembled and were manually excluded from the final MAG.

After manual refinement, the remaining contigs were individually annotated to better characterize phage-related elements. Functional annotation was performed using a database specifically curated for WO prophage genes [24]. In parallel, the origin of replication of the *w*PipCelG genome was predicted using ORI-Finder [44].

### Phylogenetic analyses

All phylogenetic analyses were performed using the Maximum Likelihood (ML) method using RAxML-NG v1.1.0 [77], and 1,000 bootstrap replicates to assess node support. The best fit amino acid substitution models were identified using ModelTest-NG v0.1.7 [78], based on the Akaike Information Criterion corrected (AICc). A ML tree was generated using concatenated nucleic sequence of six marker genes, *pk1*, *ank2*, *pk2*, *mutL*, *gp12*, and *gp15* to determine the *w*Pip group of *w*PipCelG [34]. The srWO groups of the WO prophages was assessed by constructing a ML tree based on the amino acid sequences of the large serine recombinases as defined by Bordenstein & Bordenstein (2022) [12].

### Analyses of plasmid contigs and reads

Contig_9 (containing two complete pWCP units in a direct head-to-tail tandem organization) was aligned against the full length pWCP reference sequence with Jalview [79], allowing for the identification of nucleotide level differences. As highlighted by Trigodet and colleagues, long read assemblies, particularly of circular or repeated elements, may introduce structural artefacts that can affect biological interpretation [80]. We therefore examined the corresponding raw long reads to assess the read level support for the assembled plasmid structure. Long reads mapping to contig_9 were extracted using Samtools v1.10, retaining only those with a mapping quality (MAPQ) score ≥ 2. Then, we selected only reads longer than 9,000 bp with an *in-house* awk script after which they were aligned to the reference pWCP sequence and to the dimeric contig (**see** S5 Notebook).

Because the ligation sequencing kit for gDNA involves the addition of adapters to both ends of DNA fragments for sequencing, we hypothesized there is a risk that adapters may remain ligated between two reads after sequencing, leading to the formation of chimeric sequences. To verify that this was not the case, we checked for the absence of adapters in the extracted reads with a BLAST analysis and inspection in Integrative Genomics Viewer (IGV v2.17.3) (**see** S6 Notebook).

### Identification of the origin of replication and iterons in the pWCP reference sequence

We conducted an independent analysis of the pWCP reference sequence [9] to identify replication features and investigate the potential mechanism underlying the multimeric form of the plasmid observed in our data. The putative origin of replication (oriV) was predicted using OriV-Finder for plasmids [43], a dedicated web server that integrates conserved replication initiation protein domains, iteron-like repeat motifs, and AT-rich regions to detect candidate oriV sequences.

### Long read based analysis of WO phage regions

To probe synteny and potential rearrangements in WO prophage regions directly from the sequencing data, we analyzed the raw Nanopore reads directly. From the genome-wide long read alignment to the *w*PipCelG assembly, we selected reads longer than 10,000 bp that did not map to contig_2 (the *Wolbachia* core-genome contig) nor contig_9 (the dimeric plasmid), yielding 254 long reads. Each read was annotated by nucleotide alignment against the *w*PipPel reference genome (accession number: AM999887.1) using BLASTn. We retained for each read up to five, non-overlapping high-scoring segment pairs, ranked by bit score and percent identity (>80%), and discarded lower-scoring overlaps to accommodate possible intra read structural rearrangements relative to the reference.

Then, we derived per read gene–gene adjacency counts from the annotated long reads (≥10 kb) to capture alternative gene orders underlying potential mis-assembly or assembly difficulty. For each read, WO gene hits were ordered by their coordinates on the read, and every consecutive pair was tallied as an adjacency event. Adjacency counts were then aggregated across reads to obtain edge weights and used to build a compact synteny graph centered on the Type I *cif* genes.

### Short read sequencing of *Culex pipiens molestus* ovaries

Due to the limited amount of DNA that can be extracted from a single pair of ovaries, it was not technically feasible to perform hybrid sequencing (*i.e*., both long and short read sequencing) on the same individual without prior DNA amplification. We collected and sequenced additional ovary samples from the same mosquito strain using Illumina short reads to overcome this limitation and allow for subsequent verification of candidate mutations identified in the long read data. Genomic DNA was extracted from three independent pairs of ovaries (from the same mosquito strain and using the same dissection protocol) using the DNeasy Blood & Tissue Kit (Qiagen). Library preparation and Illumina NovaSeq 6000 sequencing (2 × 150 nt, S4 flow cell) were performed by the MGX platform (Montpellier GenomiX, France) using the Illumina DNA Prep Kit. Tagmentation, indexing, quantification, and quality controls were carried out according to the manufacturer’s default protocol. Sequencing data were demultiplexed with bcl2fastq (v2.20.0.422), and quality assessed with FastQC (v0.11.9) and FastQ Screen.

### Short read-based verification of candidate mutations

The raw short reads from the three Celestin strain samples were processed using the AliNE workflow (version 1.1.0; [81]), which includes trimming with fastp, quality control with FastQC, and mapping with Bowtie2 to the newly assembled *w*PipCelG genome. Alignments were used to re-examine the mutations initially identified in the dimeric plasmid relative to the reference sequence and determine whether Nanopore-specific mutations were also supported by Illumina reads.

## Supporting information

S1 Table qPCR-based quantification of *Wolbachia*, WO and pWCP plasmid. (XLSX)

S2 Table Summary of Nanopore read assignment and filtering statistics. Number of reads, total bases, read length statistics, quality scores, and proportion of reads assigned to each category during the Nanopore data processing workflow, including host associated, lambda phage, *Wolbachia*, secondary mapping, and unassigned reads.
(XLSX)

S3 Table List of the 10 genes unique to *w*PipCelG and 8 genes not present in *w*PipCelG. (XLSX)

S4 Table Summary of contig-level assembly metrics and sequencing. This table reports the characteristics of each contig identified in the *w*PipCelG assembly, shown separately for raw mapping data (before quality filtering) and after applying a mapping quality filter of MapQ > 2. The columns indicate: BLAST_identification: best BLAST hit obtained for the contig, indicating its associated phage type when applicable. Coverage_qc_of_splits: sequencing depth across different splits of the dataset. Longest_read: length (in bp) of the longest read mapped to the contig. Mean_read_length: mean length (in bp) of the reads mapped to the contig. N50_reads: N50 value for the reads mapped to the contig, representing the read length above which 50% of the mapped bases are contained. Detection: proportion of samples or splits in which the contig was detected.
(XLSX)

S5 Table Start and end positions of pWCP-associated long reads. Start and end positions of the long reads mapped to pWCP plasmid.
(XLSX)

S6 Table PFAM annotation of the *w*PipCelG genome. GFF annotation file of the *w*PipCelG assembly containing predicted coding sequences and associated PFAM domain annotations.
(GFF)

S7 Table Details of sample preparation for long-read sequencing. (XLSX)

S1 Fig Phylogenetic placement of *w*PipCelG within the *w*Pip groups. Maximum Likelihood phylogeny inferred from the concatenated alignment of six genes (*mutL*, *ank2*, *pk1*, *pk2*, *GP12*, and *GP15*) totaling 5,423 bp. Bootstrap values were estimated using 1,000 replicates. The tree was constructed using the GTR + I substitution model.
(PDF)

S2 Fig Pangenome with *w*PipCelG, *w*PipPel, *w*PipJHB and *w*PipMol. A total of 1,166 gene clusters were identified and hierarchically clustered in the dendrogram based on their presence/absence patterns. The heatmap displays, from top to bottom: ANIm alignment nucleotide identity, number of gene clusters, number of singleton gene clusters, number genes by kbp, GC content, and total genome length.
(PDF)

S3 Fig Sequence alignment between the dimeric pWCP contig of *w*PipCelG and the reference pWCP. The dimeric plasmid contig was split into two monomeric units (positions 1–9,242 and 9,243–18,485) and aligned independently to the reference pWCP to assess sequence identity and confirm the dimeric configuration.
(PDF)

S4 Fig Hypothetical model of theta-replication in the pWCP plasmid. (A) Representative reads were mapped to the monomeric pWCP reference sequence (B) Schematic interpretation of forward and reverse strand potentially from a single DNA molecule undergoing theta replication (C) Example of a plasmid read containing overlapping sense and antisense regions consistent with replication fork progression.
(PDF)

S5 Fig Identification of a putative origin of replication (oriV) in the pWCP plasmid. The oriV region was predicted using OriV-Finder [43] and is located between positions 6,363 and 6,513 bp of the pWCP sequence. This region overlaps with a tandem repeat zone previously annotated as a VNTR and contains a set of putative iterons (dark brown), characteristic of iteron-based replication initiation mechanisms.
(PNG)

S6 Fig Phylogeny on the serine recombinase of WO. Maximum Likelihood tree inferred from amino acid sequences of large serine recombinases (LSRs) encoded by WO prophages, including sequences from the reference *w*Mel genome (WD_0288 and WD_0634) and representative members of the four srWO clades defined by Bordenstein & Bordenstein (2022). Newly identified sequences from this study are highlighted in red. Node support values represent bootstrap percentages (1,000 replicates), and the tree is midpoint-rooted.
(PDF)

S7 Fig Coverage of filtered reads mapped to phage WO genes clusters and estimation of gene copy numbers. Filtered reads were mapped to representative sequences of phage WO gene clusters, and coverage depth is shown alongside estimated gene copy numbers in *w*PipCelG (in red), assembled copies in *w*PipCelG (in green), and in *w*PipPel (in blue). Gene clusters were generated during pangenomic analyses, and each coverage value corresponds to the mapping depth on the longest gene of the cluster. The figure aggregates all modules, including integration/replication (transposases, ankyrin repeats, connector/baseplate, replication/repair), structural and effector components (tail, head, tail fiber, toxins, cytoplasmic incompatibility factors), and additional modules of interest (repair machinery, transcriptional regulators, DUF1016, *WO-PC1*, patatin phospholipases, chaperones, metabolism-related genes, and six remaining individual genes). The average estimated copy number per module (red value) is indicated in the upper-right inset of each panel.
(PDF)

S8 Fig The *cifA* (Type I) synteny puzzle: long-read gene-adjacency graph. Directed gene-adjacency graph derived from 60 annotated Nanopore reads (≥10 kb) that contained *cif (*Type I or IV) and/or genes from the region near these loci (*e.g.*, transposases WP0281/WP0291, *rnhA* WP0279, *mutL*WP0278, *intII* WP0280, TR/wmk-like). For each long read, WO gene hits were ordered by their coordinates on the read, and every consecutive pair was tallied as an adjacency event. Nodes represent genes; edges represent observed adjacencies, labeled with the number of adjacency events aggregated across reads, and drawn with widths proportional to this count. The layout is rooted at *cifA* (WP0282) to emphasize alternative routes emanating from this gene.
(TIFF)
