# Supplementary material for: Extensive mobilome heterogeneity in a widespread endosymbiont: A candidate dimeric plasmid and highly fragmented prophages in Wolbachia from Culex pipiens
Source: PLoS Genet. 2026 Oct 26;22(10):e1012347. doi: 10.1371/journal.pgen.1012347 (PMC13734603; doi:10.1371/journal.pgen.1012347)
Supplement: Supplementary file 1 [file pgen.1012347.s008.pdf]

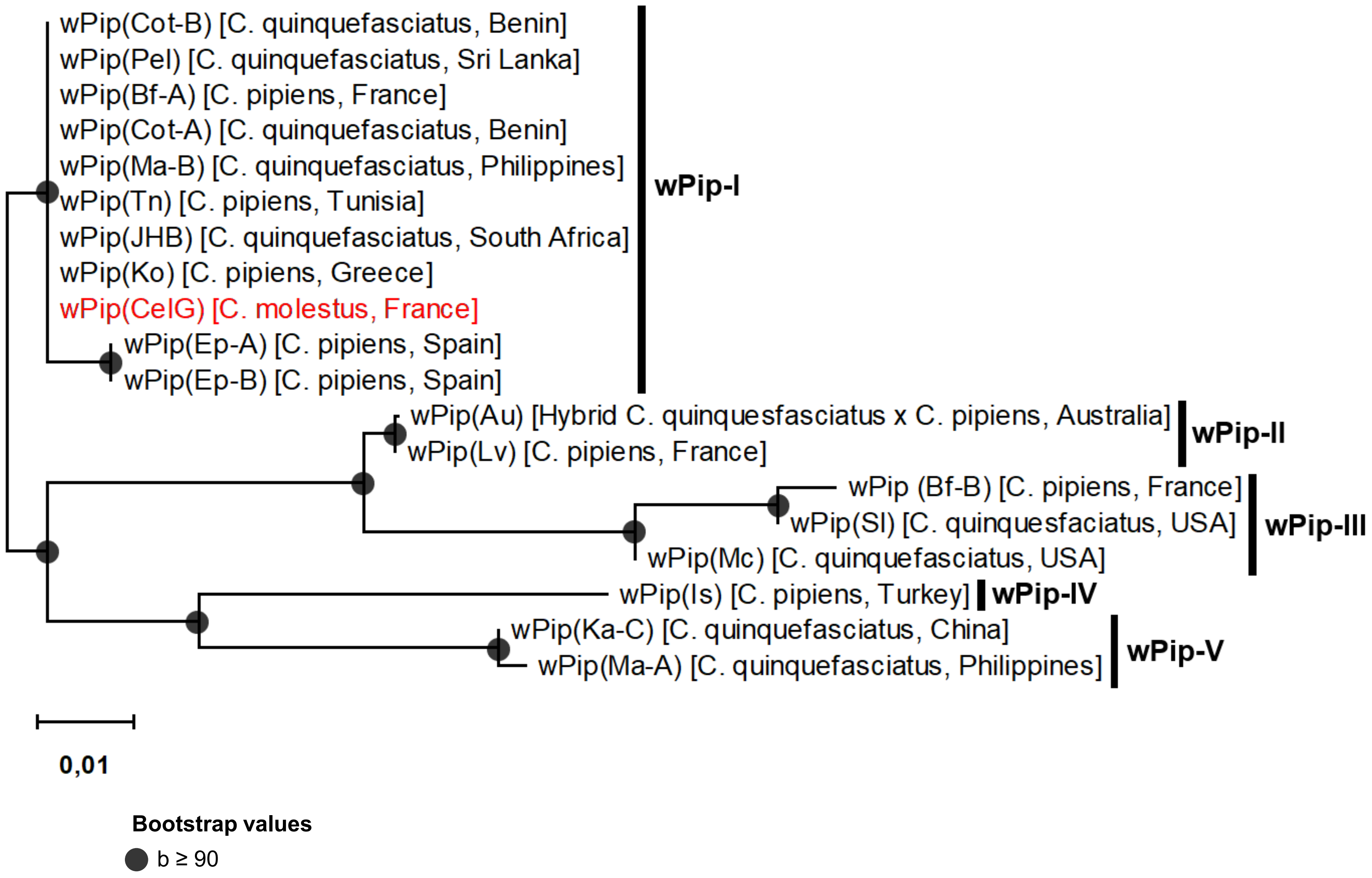
wPip(Cot-B) [C. quinquefasciatus, Benin]
wPip(Pel) [C. quinquefasciatus, Sri Lanka]
wPip(Bf-A) [C. pipiens, France]
wPip(Cot-A) [C. quinquefasciatus, Benin]
wPip(Ma-B) [C. quinquefasciatus, Philippines]
wPip(Tn) [C. pipiens, Tunisia]
wPip(JHB) [C. quinquefasciatus, South Africa]
wPip(Ko) [C. pipiens, Greece]
wPip(CelG) [C. molestus, France]
wPip(Ep-A) [C. pipiens, Spain]
wPip(Ep-B) [C. pipiens, Spain]
wPip-I
wPip(Au) [Hybrid C. quinquesfasciatus x C. pipiens, Australia]
wPip(Lv) [C. pipiens, France]
wPip-II
wPip (Bf-B) [C. pipiens, France]
wPip(Sl) [C. quinquesfaciatus, USA]
wPip(Mc) [C. quinquefasciatus, USA]
wPip-III
wPip(Is) [C. pipiens, Turkey]
wPip-IV
wPip(Ka-C) [C. quinquefasciatus, China]
wPip(Ma-A) [C. quinquefasciatus, Philippines]
wPip-V
0,01
Bootstrap values
b ≥ 90
