# Supplementary material for: Extensive mobilome heterogeneity in a widespread endosymbiont: A candidate dimeric plasmid and highly fragmented prophages in Wolbachia from Culex pipiens
Source: PLoS Genet. 2026 Oct 26;22(10):e1012347. doi: 10.1371/journal.pgen.1012347 (PMC13734603; doi:10.1371/journal.pgen.1012347)
Supplement: Supplementary file 2 [file pgen.1012347.s009.pdf]

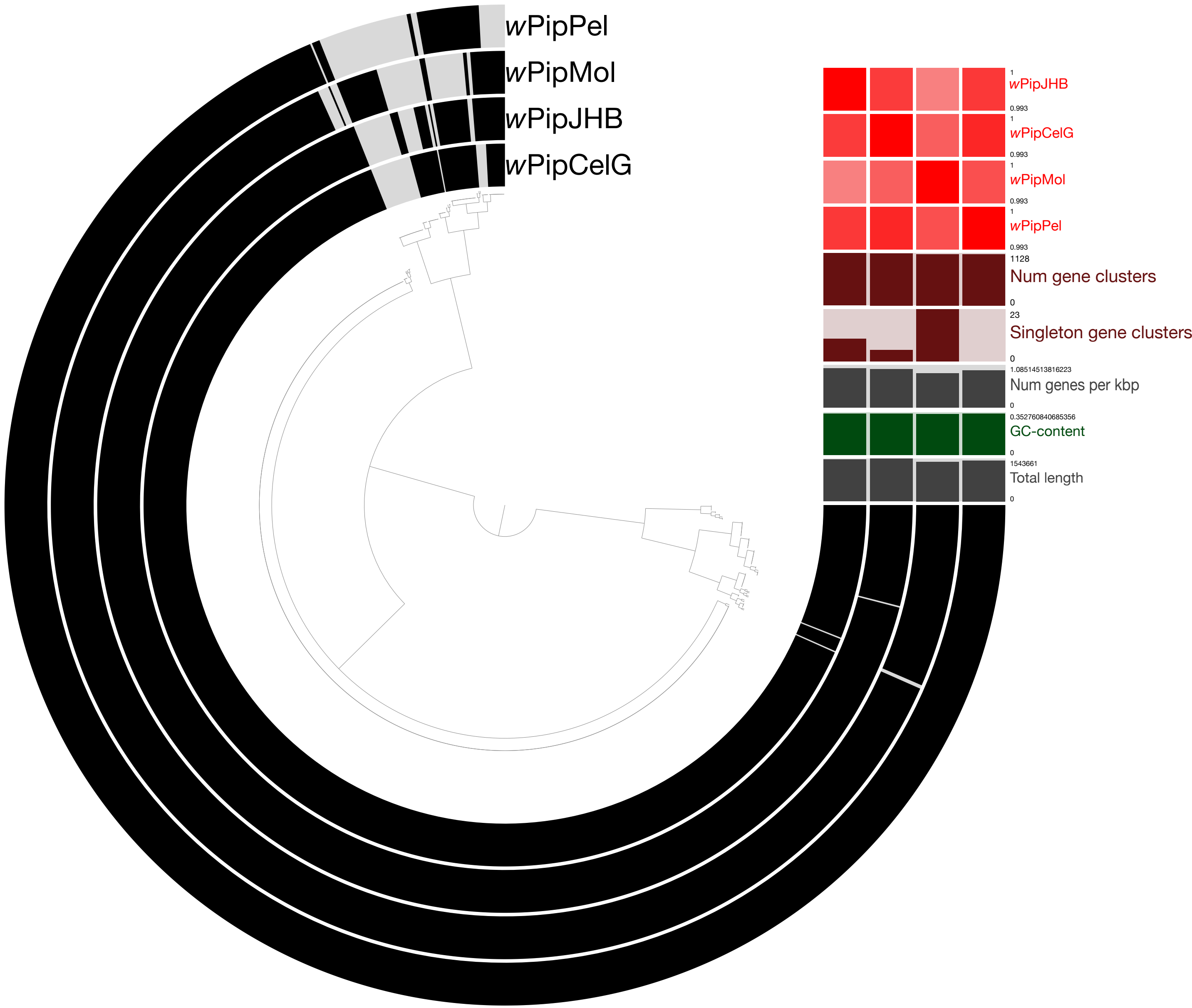

*w*PipPel
*w*PipMol
*w*PipJHB
*w*PipCelG
1
*w*PipJHB
0.993
1
*w*PipCelG
0.993
1
*w*PipMol
0.993
1
*w*PipPel
0.993
1128
Num gene clusters
0
23
Singleton gene clusters
0
1.08514513816223
Num genes per kbp
0
0.352760840685356
GC-content
0
1543661
Total length
0
