# Supplementary material for: Extensive mobilome heterogeneity in a widespread endosymbiont: A candidate dimeric plasmid and highly fragmented prophages in Wolbachia from Culex pipiens
Source: PLoS Genet. 2026 Oct 26;22(10):e1012347. doi: 10.1371/journal.pgen.1012347 (PMC13734603; doi:10.1371/journal.pgen.1012347)
Supplement: Supplementary file 3 [file pgen.1012347.s010.pdf]

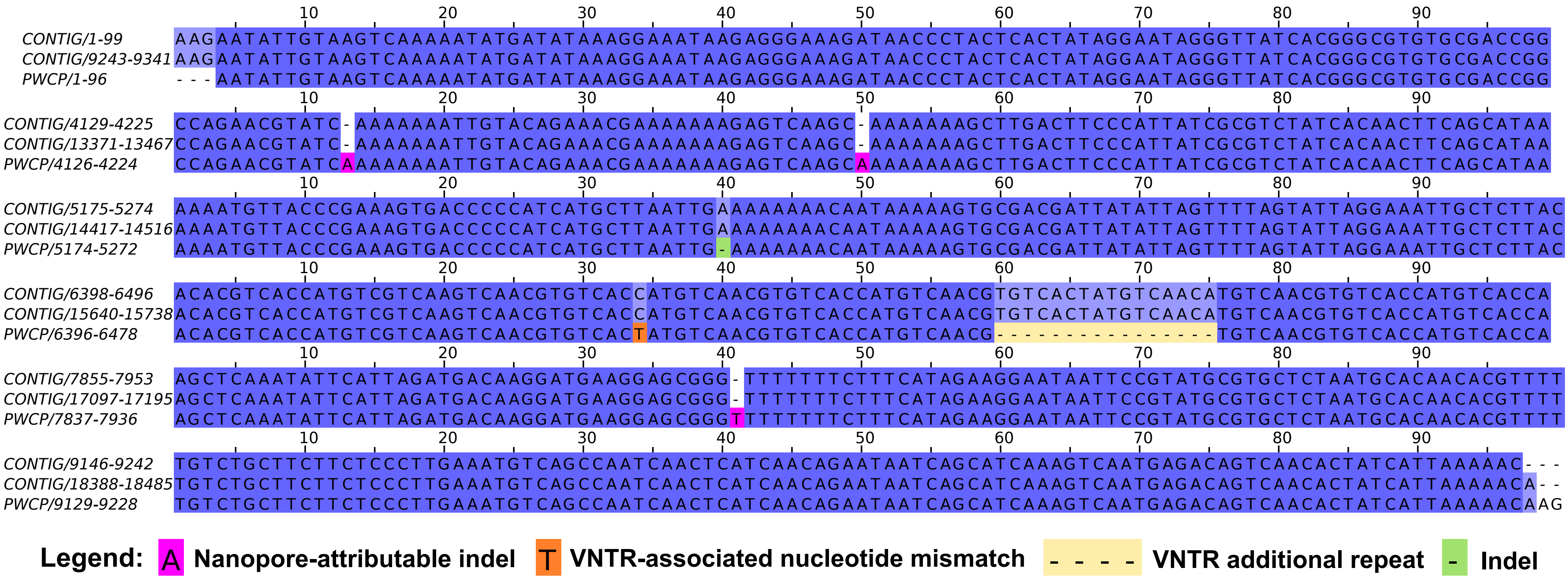
CONTIG/1-99
CONTIG/9243-9341
PWCP/1-96
CONTIG/4129-4225
CONTIG/13371-13467
PWCP/4126-4224
CONTIG/5175-5274
CONTIG/14417-14516
PWCP/5174-5272
CONTIG/6398-6496
CONTIG/15640-15738
PWCP/6396-6478
CONTIG/7855-7953
CONTIG/17097-17195
PWCP/7837-7936
CONTIG/9146-9242
CONTIG/18388-18485
PWCP/9129-9228
Legend:
A Nanopore-attributable indel
T VNTR-associated nucleotide mismatch
- - - - VNTR additional repeat
- Indel
