# Supplementary material for: Extensive mobilome heterogeneity in a widespread endosymbiont: A candidate dimeric plasmid and highly fragmented prophages in Wolbachia from Culex pipiens
Source: PLoS Genet. 2026 Oct 26;22(10):e1012347. doi: 10.1371/journal.pgen.1012347 (PMC13734603; doi:10.1371/journal.pgen.1012347)
Supplement: Supplementary file 4 [file pgen.1012347.s011.pdf]

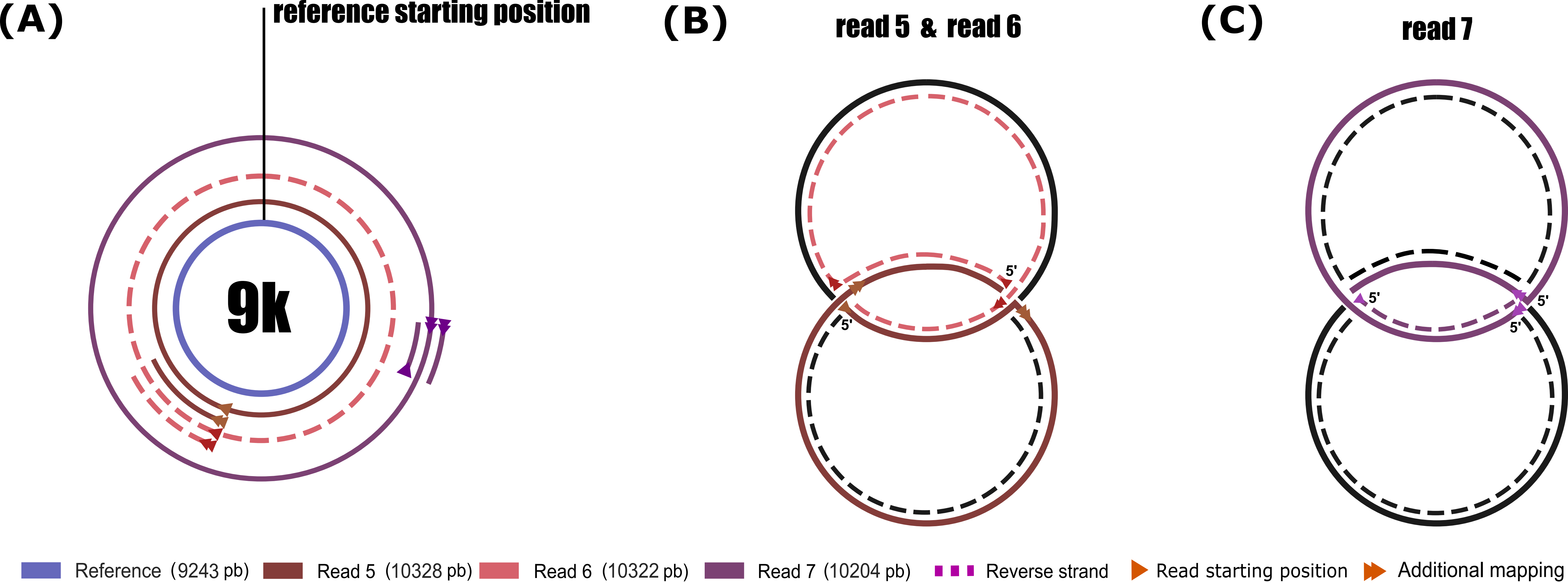

(A)
reference starting position
9k
(B)
read 5 & read 6
5'
5'
(C)
read 7
5'
5'
5'
Reference (9243 pb)
Read 5 (10328 pb)
Read 6 (10322 pb)
Read 7 (10204 pb)
Reverse strand
Read starting position
Additional mapping
