# Supplementary material for: Extensive mobilome heterogeneity in a widespread endosymbiont: A candidate dimeric plasmid and highly fragmented prophages in Wolbachia from Culex pipiens
Source: PLoS Genet. 2026 Oct 26;22(10):e1012347. doi: 10.1371/journal.pgen.1012347 (PMC13734603; doi:10.1371/journal.pgen.1012347)
Supplement: Supplementary file 5 [file pgen.1012347.s013.pdf]

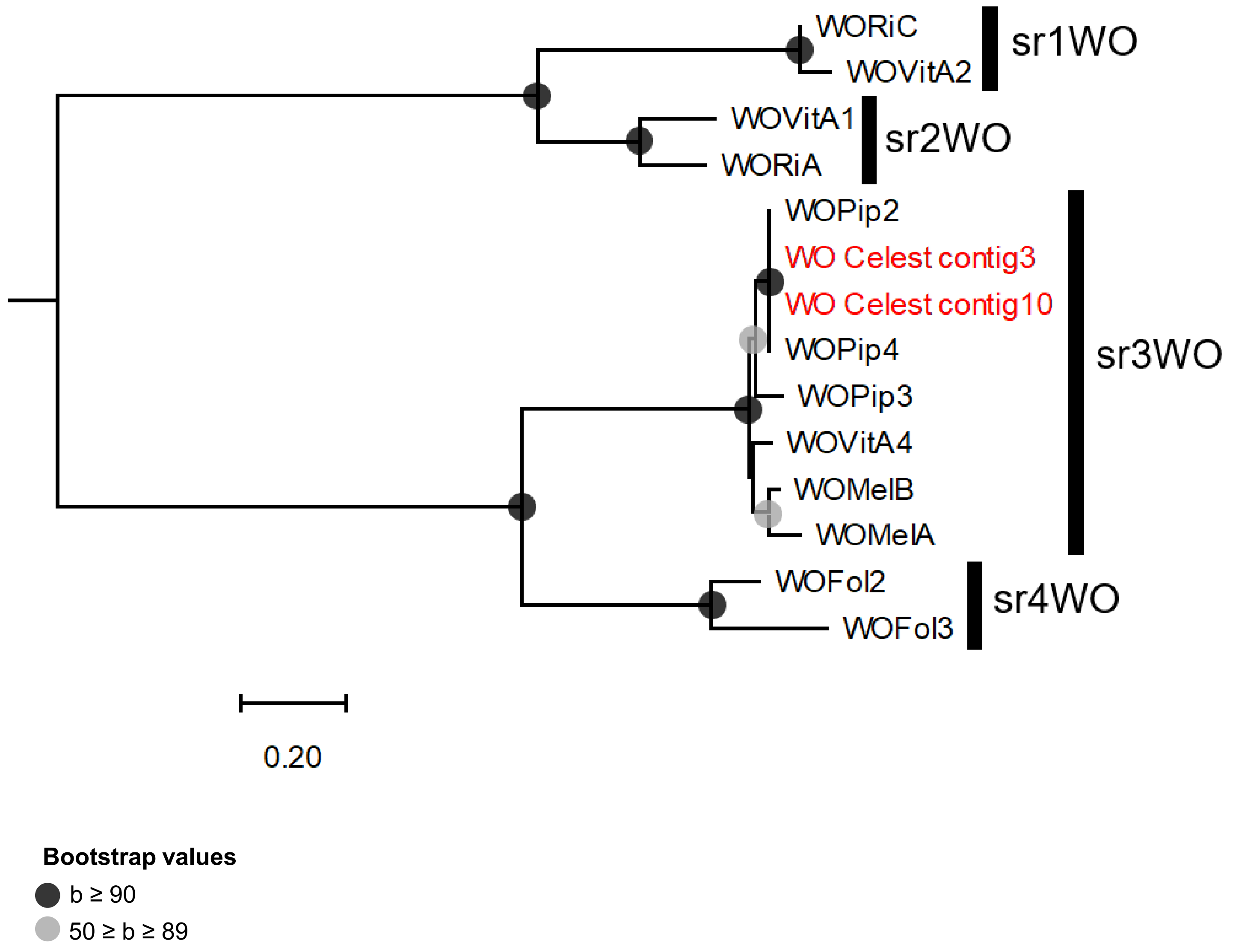

WORiC
WOVitA2
sr1WO
WOVitA1
WORiA
sr2WO
WOPip2
WO Celest contig3
WO Celest contig10
WOPip4
WOPip3
WOVitA4
WOMelB
WOMelA
sr3WO
WOFol2
WOFol3
sr4WO
0.20
Bootstrap values
b ≥ 90
50 ≥ b ≥ 89
