# Supplementary material for: Extensive mobilome heterogeneity in a widespread endosymbiont: A candidate dimeric plasmid and highly fragmented prophages in Wolbachia from Culex pipiens
Source: PLoS Genet. 2026 Oct 26;22(10):e1012347. doi: 10.1371/journal.pgen.1012347 (PMC13734603; doi:10.1371/journal.pgen.1012347)
Supplement: Supplementary file 6 [file pgen.1012347.s014.pdf]

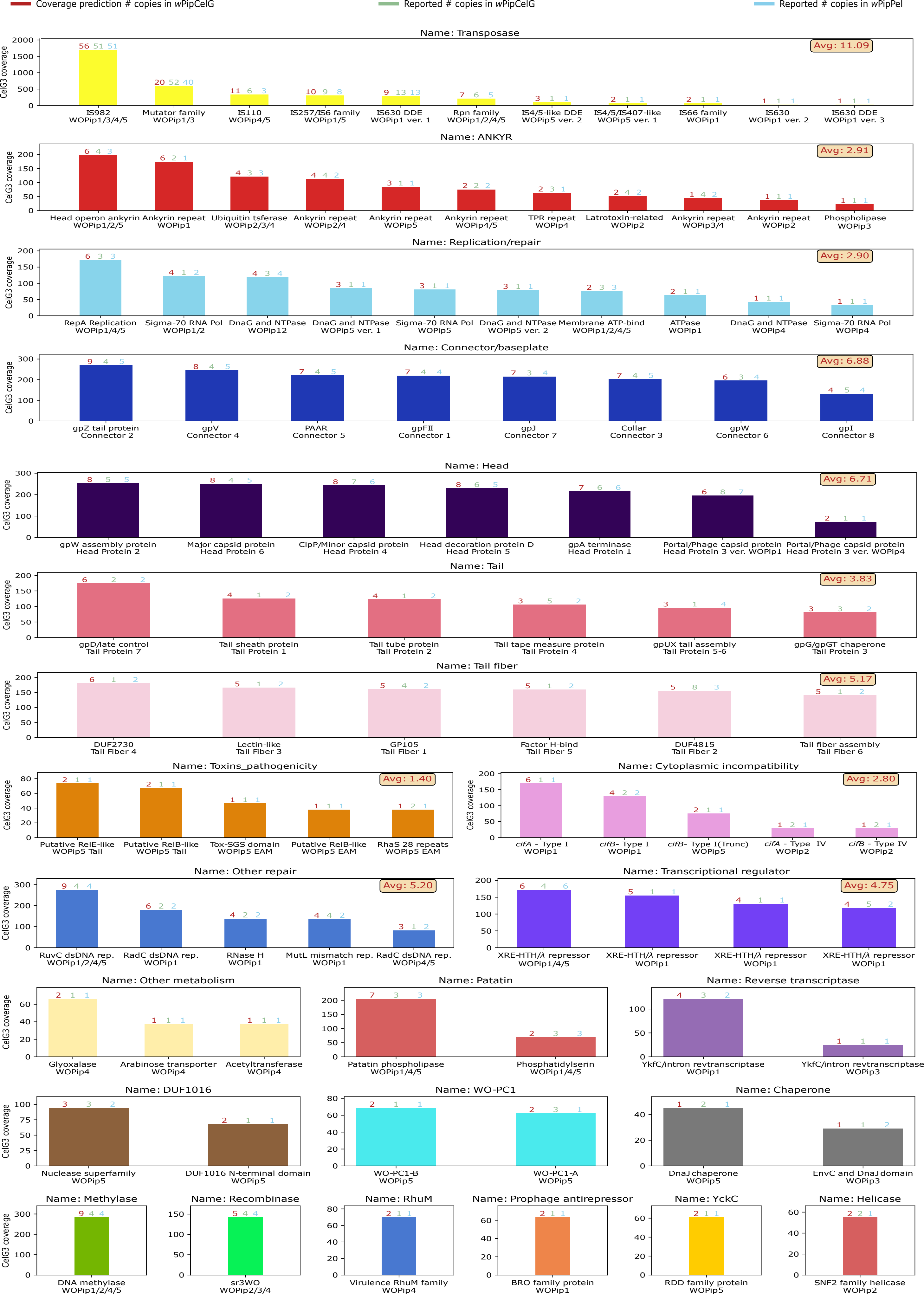

Coverage prediction # copies in wPipCelG
Reported # copies in wPipCelG
Reported # copies in wPipPel
CelG3 coverage
Name: Transposase
Avg: 11.09
56 51 51 IS982 WOPip1/3/4/5
20 52 40 Mutator family WOPip1/3
11 6 3 IS110 WOPip4/5
10 9 8 IS257/IS6 family WOPip1/5
9 13 13 IS630 DDE WOPip1 ver. 1
7 6 5 Rpn family WOPip1/2/4/5
3 1 1 IS4/5-like DDE WOPip5 ver. 2
2 1 1 IS4/5/IS407-like WOPip5 ver. 1
2 1 1 IS66 family WOPip1
1 1 1 IS630 WOPip1 ver. 2
1 1 1 IS630 DDE WOPip1 ver. 3
Name: ANKYR
Avg: 2.91
6 4 3 Head operon ankyrin WOPip1/2/5
6 2 1 Ankyrin repeat WOPip1
4 3 3 Ubiquitin tsferase WOPip2/3/4
4 4 2 Ankyrin repeat WOPip2/4
3 1 1 Ankyrin repeat WOPip5
2 2 2 Ankyrin repeat WOPip4/5
2 3 1 TPR repeat WOPip4
2 4 2 Latrotoxin-related WOPip2
1 4 2 Ankyrin repeat WOPip3/4
1 1 1 Ankyrin repeat WOPip2
1 1 1 Phospholipase WOPip3
Name: Replication/repair
Avg: 2.90
6 3 3 RepA Replication WOPip1/4/5
4 1 2 Sigma-70 RNA Pol WOPip1/2
4 3 4 DnaG and NTPase WOPip12
3 1 1 DnaG and NTPase WOPip5 ver. 1
3 1 1 Sigma-70 RNA Pol WOPip5
3 1 1 DnaG and NTPase WOPip5 ver. 2
2 3 3 Membrane ATP-bind WOPip1/2/4/5
2 1 1 ATPase WOPip1
1 1 1 DnaG and NTPase WOPip4
1 1 1 Sigma-70 RNA Pol WOPip4
Name: Connector/baseplate
Avg: 6.88
9 4 5 gpZ tail protein Connector 2
8 4 5 gpV Connector 4
7 4 5 PAAR Connector 5
7 4 4 gpFII Connector 1
7 3 4 gpJ Connector 7
7 4 5 Collar Connector 3
6 3 4 gpW Connector 6
4 5 4 gpI Connector 8
Name: Head
Avg: 6.71
8 5 5 gpW assembly protein Head Protein 2
8 4 5 Major capsid protein Head Protein 6
8 7 6 ClpP/Minor capsid protein Head Protein 4
8 6 5 Head decoration protein D Head Protein 5
7 6 6 gpA terminase Head Protein 1
6 8 7 Portal/Phage capsid protein Head Protein 3 ver. WOPip1
2 1 1 Portal/Phage capsid protein Head Protein 3 ver. WOPip4
Name: Tail
Avg: 3.83
6 2 2 gpD/late control Tail Protein 7
4 1 2 Tail sheath protein Tail Protein 1
4 1 2 Tail tube protein Tail Protein 2
3 5 2 Tail tape measure protein Tail Protein 4
3 1 4 gpUX tail assembly Tail Protein 5-6
3 3 2 gpG/gpGT chaperone Tail Protein 3
Name: Tail fiber
Avg: 5.17
6 1 2 DUF2730 Tail Fiber 4
5 1 2 Lectin-like Tail Fiber 3
5 4 2 GP105 Tail Fiber 1
5 1 2 Factor H-bind Tail Fiber 5
5 8 3 DUF4815 Tail Fiber 2
5 1 2 Tail fiber assembly Tail Fiber 6
Name: Toxins_pathogenicity
Avg: 1.40
2 1 1 Putative RelE-like WOPip5 Tail
2 1 1 Putative RelB-like WOPip5 Tail
1 1 1 Tox-SGS domain WOPip5 EAM
1 1 1 Putative RelB-like WOPip5 EAM
1 2 1 RhaS 28 repeats WOPip5 EAM
Name: Cytoplasmic incompatibility
Avg: 2.80
6 1 1 cifA - Type I WOPip1
4 2 2 cifB- Type I WOPip1
2 1 1 cifB- Type I(Trunc) WOPip5
1 2 1 cifA - Type IV WOPip2
1 2 1 cifB - Type IV WOPip2
Name: Other repair
Avg: 5.20
9 4 4 RuvC dsDNA rep. WOPip1/2/4/5
6 2 2 RadC dsDNA rep. WOPip1
4 2 2 RNase H WOPip1
4 4 2 MutL mismatch rep. WOPip1
3 1 2 RadC dsDNA rep. WOPip4/5
Name: Transcriptional regulator
Avg: 4.75
6 4 6 XRE-HTH/λ repressor WOPip1/4/5
5 1 1 XRE-HTH/λ repressor WOPip1
4 1 1 XRE-HTH/λ repressor WOPip1
4 5 2 XRE-HTH/λ repressor WOPip1
Name: Other metabolism
2 1 1 Glyoxalase WOPip4
1 1 1 Arabinose transporter WOPip4
1 1 1 Acetyltransferase WOPip4
Name: Patatin
7 3 3 Patatin phospholipase WOPip1/4/5
2 3 3 Phosphatidylserin WOPip1/4/5
Name: Reverse transcriptase
4 3 2 YkfC/intron revtranscriptase WOPip1
1 1 1 YkfC/intron revtranscriptase WOPip3
Name: DUF1016
3 3 2 Nuclease superfamily WOPip5
2 1 1 DUF1016 N-terminal domain WOPip5
Name: WO-PC1
2 1 1 WO-PC1-B WOPip5
2 3 1 WO-PC1-A WOPip5
Name: Chaperone
1 2 1 DnaJ chaperone WOPip5
1 1 2 EnvC and DnaJ domain WOPip3
Name: Methylase
9 4 4 DNA methylase WOPip1/2/4/5
Name: Recombinase
5 4 4 sr3WO WOPip2/3/4
Name: RhuM
2 1 1 Virulence RhuM family WOPip4
Name: Prophage antirepressor
2 1 1 BRO family protein WOPip1
Name: YckC
2 1 1 RDD family protein WOPip5
Name: Helicase
2 2 1 SNF2 family helicase WOPip2
