# Supplementary material for: Extensive mobilome heterogeneity in a widespread endosymbiont: A candidate dimeric plasmid and highly fragmented prophages in Wolbachia from Culex pipiens
Source: PLoS Genet. 2026 Oct 26;22(10):e1012347. doi: 10.1371/journal.pgen.1012347 (PMC13734603; doi:10.1371/journal.pgen.1012347)
Supplement: Supplementary file 7 [file pgen.1012347.s016.pdf]

Overall assessment

This is an interesting, thoughtful, and potentially valuable study. The amplification-free, single-ovary ONT strategy is well matched to the biological question, and the manuscript is generally clear, well organized, and thoughtfully analyzed. In particular, the careful inspection of raw reads, the caution around assembly limitations, and the well-documented computational notebooks are appreciated. The study provides a useful high-contiguity wPip genome, a nice proof of concept for applying long-read sequencing to low-biomass, host-rich *Wolbachia* samples, and some intriguing new evidence for wPip's potential mobilome dynamics; overall it lays useful groundwork for future studies to build more knowledge in this exciting area.

I have several recommendations that I hope will help strengthen the manuscript, and that I hope the authors will find helpful. First, the study would benefit from clearer reporting of the sequencing, filtering, assembly, and contig-curation decisions, since these choices are central to interpreting the results. Second, while the manuscript is valuable as-is, some small additional analysis and contextualization would make the broader claims about mobilome dynamics and MGE activity more convincing.

Major comments

Sequencing strategy, carrier DNA, possible artifacts

The sequencing approach (single ovary, amplification-free, lambda carrier spike-in) is interesting and seems to make sense. As it is uncommon/novel and central to the manuscript, the decisions should be justified and discussed more explicitly. For example, the rationale for avoiding PCR is reasonable, but the manuscript would benefit from a clearer explanation of the tradeoffs involved, including advantages of avoiding amplification, possible biases introduced by carrier DNA, and placing these approach decisions in context of the recent literature (e.g. other low-biomass microbiome study approaches or precedent for using carrier DNA).

A more complete summary of read filtering and read assignment would also improve confidence in the downstream analyses. In particular, it would be helpful to report more stats e.g. reads/bases assigned to host/lambda (& filtered out), Wolbachia chromosomal regions, plasmid regions, WO-rich contigs, low-confidence/multimapping categories, and unassigned categories. This is especially important because some central conclusions depend on relatively small numbers of informative long reads. Maybe a flowchart schematic would be helpful.

The direct analysis of raw long reads nicely avoids potential assembly artifacts. However, ligation-based ONT libraries can still generate chimeric molecules (the authors mention this briefly in the Discussion; some evidence it can be ~1%). The current adapter-checking step is useful, but the manuscript should more directly address whether host/lambda/Wolbachia split-read artifacts could affect filtering or interpretation. For example, during the filtering step, reads could be screened/classified for split alignments, rather than just their best-hit taxonomic assignment, to see whether this is an issue. I don't know if this will turn anything up and don't expect it to affect your conclusions, but would be a good due diligence to

increase confidence in the results, since they rely on direct inspection of a small number of raw reads.

Specific points:

- [154] Accordingly, please soften the claim that the sequencing strategy avoids chimeric sequences. Avoiding PCR reduces one source of chimerism, but ligation-based long-read sequencing can still generate chimeric reads. The later discussion around 428–434 is more cautious and could be made consistent with the earlier wording.
- [Methods] Please cite or briefly discuss precedent and tradeoffs for lambda carrier DNA in low-input sequencing; state the flow cell details and pore chemistry; clarify how atypical contigs likely occurred.

Assembly parameters and contig curation

The author's approach of manual contig curation and avoiding over-interpretation of the assembly are generally reasonable, but the manuscript should provide more detail about the origin of the excluded contigs and the robustness of the final assembly.

The contigs with negligible coverage or 100% identity to internal regions of larger contigs (line 202-209) are somewhat surprising to me, especially for recent versions of Flye (unlike e.g. older versions of CANU where I might expect some of this more). It would be useful to provide more information for accounting of these contigs — whether they are low-support contigs, multimapping/repeat-derived contigs, residual host-like sequence (e.g. related to *Culex* masking choices and EAM phage regions), or artifacts related to assembly and filtering choices (detailed below).

Because the highly contiguous wPip genome is one of the study's main outputs, the assembly parameters should be reported directly in the Methods, including Flye version and key options. It would also be helpful to briefly state whether alternative common assembly settings were considered and whether they affected the main chromosome, plasmid, or WO-rich contigs. If the authors have not already considered, I suggest several specific recommendations here:

1. Run Flye filtered only to longer reads (maybe >2 kb), and include the —keep-haplotypes flag. This may help improve assembly contiguity and the ability to resolve the possible phage structural variants.
2. In general the —nano-raw flag is only recommended for older technology (higher error rate and older Guppy). Referring to the Flye usage docs on GitHub, using —nano-hq for Guppy5+ SUP and adding —read-error .05 seems to be the recommended choice based on the data here.
3. The —meta mode was probably the correct choice (as noted by the authors in the workbook), but it might be worth running Flye without it to see if this improves chromosome or plasmid contiguity at all.

The existing parameter choices may already be optimal for this particular dataset, but the authors should consider, test, and justify their parameter choices, given that the resulting genome is a key contribution of the study.

pWCP plasmid dimerization & replication evidence

The pWCP results are compelling, but the interpretation should more clearly separate observed structures from proposed mechanisms.

The evidence for monomeric and dimeric/multimeric pWCP forms should be presented separately from the evidence for active replication. A dimeric plasmid, a replication intermediate, an unresolved recombination product, and a concatemer are related but distinct possibilities. The manuscript discusses these possibilities, but the Results and Discussion would be clearer if the various observed read/contig structures were distinguished more explicitly from each other and from the mechanistic interpretation.

It would also help to clarify how the 9 kb and 18 kb forms relate to one another structurally, especially around the proposed oriV/VNTR region and what the inferred dimer junction looks like (is the 18 kb form a "fused plasmid" via IS/transposon-mediated recombination? does the 9kb form split near the oriV? etc.)

For example, on line 239-240, please clarify whether the shorter reads uniquely support the 9 kb monomeric form or are also consistent with reads sampled from larger circular or multimeric structures but are just not long enough to know. If the 18 kb form differs from the 9kb form near the putative oriV or at other junctions, it would be interesting and helpful information to provide.

WO phage integration, structural heterogeneity, potential activity

The WO phage results are exciting, but the interpretation would be stronger with more direct read support for integration sites and structural relationships among WO-rich contigs.

The current data support substantial under-assembled WO diversity, but several models remain possible: multiple chromosomal prophage loci, tandem structures, relic fragments, episomal or lytic phage, or rare within-host structural variants (though I'm not very familiar with what has already been specifically established about WO phage biology/genetics). The manuscript would benefit from a clearer distinction among these possibilities and, where possible, stronger read-level evidence linking WO-rich contigs to their potential chromosomal context. I suggest some small additional read-level analysis which could provide more compelling evidence for phage dynamics:

- [296] "observed core genes flanking EAM regions in almost all contigs" — where are these core genes located on contig_2? This could provide evidence for the putative phage integration site(s).
- [301/315] The authors note that the cif and Octomom-like sequence at contig_2 edges strongly suggest a phage-associated integration region. More explicit read-level support would strengthen this interpretation: are there any reads mapping to the ends of contig 2 which span across this putative phage integration site? And/or do the ends of any phage contigs (especially the ones with more uniform coverage) map back to contig_2? This could also be done at an individual read level. The Flye —keep-haplotypes flag (or running a tool like Sniffles2) could also be helpful here.
- [309] "These values suggest that the genome contains multiple intact and relic prophage elements…" — I find this to be an exciting result that's worth digging into a bit more.

    - Can the authors provide confident evidence that the sr3WO LSRs on contig_3 and contig_10 are genuinely different (rather than reflecting ONT error, collapsed repeats, or assembly ambiguity)? If so, this provides another piece of evidence to support the claim that the genome contains multiple prophage elements.
    - If there are multiple prophage elements, they are either in tandem or they have multiple integration sites in the chromosome. Furthermore, for a given chromosome, if there is read support for any chromosomal loci both with and without WO region integrated, this would heavily suggest phage activity.
    - Either way, please clarify whether the evidence supports multiple loci, tandem organization, episomal/lytic phage, or some combination.
- [395] "highly repetitive nature of prophage regions and possibly the presence of active phage particles". Please distinguish evidence for assembly fragmentation from evidence for active phage particles; more light could be shed on this by looking at read ends.

From the existing results presented in this section, I'm not convinced that the authors have provided enough evidence to claim "active" phage "dynamics" here. If the authors feel that any additional analyses would be out of scope for this manuscript, that's okay, but in that case, I think they should soften their claims about phage dynamics a bit. The existing results are intriguing and consistent with phage activity, but also potentially consistent with other possibilities. It's possible that some small additional read-level analysis would provide additional evidence for phage dynamics.

Inference of MGE activity and mobilome dynamics

The framing of an active, dynamic *Wolbachia* mobilome is plausible, but

- [Title] I'm admittedly a bit hesitant about the word "dynamics" here, though it's reasonable if the manuscript makes clear that some inferred dynamics come from structural heterogeneity rather than direct temporal observation.
- [352] More cautious wording such as "putatively active," "structurally heterogeneous," or "consistent with activity" may be more appropriate depending on the specific claim. I'm not strongly against "active MGE" but would carefully consider the conclusions supported by the data.
- The interpretation of within-host structural heterogeneity would benefit from a clearer discussion of possible *Wolbachia* strain heterogeneity within a single organ. I don't have domain knowledge here, but my understanding is that although within-host *Wolbachia* populations are often comprised of multiple strains, previous work by this group has shown there is remarkably little strain diversity for wPip in *C. pipens* specifically. The authors could even provide evidence in these data for low intra-individual diversity (e.g. Strainy on the existing assembly graph, or InStrain from the short-read data, etc.).
    - This would increase support for the authors' claim that what we're observing here is structural heterogeneity against a single genomic background, rather than strain heterogeneity.

Minor general comments

- The introduction mentions small MGEs such as insertion sequences and transposons. Given the nature of the long read data generated here, it might be interesting to briefly comment on any observed IS/transposon heterogeneity or putative activity in the chromosome (other than what was found on WOPip5).
- The Discussion could briefly mention how newer ONT chemistry (R10.4.1), basecalling (Dorado), and adaptive sampling approaches (to preferentially reject host & lambda molecules) might improve future work on similar low-biomass, host-rich samples.

Minor line-by-line comments

- [155] The term “punctual mutation” may be more of a French-specific convention; I’d recommend “point mutation ” (or SNV depending on the intended meaning)
- [174] “Same strain” appears to refer to the mosquito strain, but this could be confused with *Wolbachia* strain. Please ensure that the intended meaning is clear (here and several other places in the manuscript).
- [Fig. 1] Consider adding anatomical labels (for readers less familiar, given the journal audience). Relatedly, some additional background on WO phage biology might be helpful for readers unfamiliar with the domain.
- [278] “Unassembled prophage regions” could be misread as unassembled reads. Please clarify whether this refers to fragmented assembly of prophage loci.
- [294] Please clarify whether “the 13 contigs containing prophage WO related regions” refers to contig_2 plus the 12 smaller WO-rich contigs.
- [Fig. 2B / S4C] The path of read 7 is difficult to follow, especially because antisense sequence appears represented differently between panels. Please harmonize or explain the visual conventions.
- [Fig. 2B] Please check consistency of the pWCP reference length. The text gives 9,228 bp, while Fig. 2 appears to show 9,243 bp.
- [Fig. S6] — overall highly informative; maybe worth having as a main figure
  - The red/phage annotation is a bit difficult for me to interpret. Please clarify whether this reflects specific prophage ORF regions, manual assignment of phage-like contigs, or another criterion.
  - Consider visually separating the small WO-rich contigs from the chromosomal representation in the Circos plot, as their order/position are not confirmed.
  - Consider calling contig_2 “chromosomal” rather than “core genome”, unless “core genome” is intended in a specific comparative-genomic sense.
  - Please add numeric labels to the phage contigs in A, corresponding to the numbering in B.
  - There are two different grey gene shadesin B, but they’re not labeled (is light grey core genes?)

- [Fig. 4A]
  - “Read 7” and “read 8” may be confused with plasmid reads 7 and 8 in Fig. 2B. Consider using more consistent read identifiers.
  - Where do those reads map to on the contigs shown in S6A/B? I understand the conclusion is that these regions are likely rearranged for wPipCel vs wPipPel, but how read 7 and read 8 map onto WOPip1/WOPip5 and how those map onto the corresponding locations in wPipCel and wPipPel genome is not clear to me.
- [Fig. 4B] Palindrome 1 is supported by 8 reads, but based on what’s shown, it seems like some of these 8 reads do not span the entire region? Consider showing a diagram (or igv-style plot) to clarify, for each of these palindromic regions, exactly where the read support lies across the region.
  - Not sure if I’m interpreting this correctly, but could read 2 map well (anti-sense) to palindrome 2 if intII was mobilized? It’s a bit hard to make sense of what might be happening here.
  - I’m generally skeptical of something that has only 1 read supporting it, but I think it’s fine to show because the authors are transparent about this.
- Figures generally: Consider using consistent colors for functional categories across figures (e.g., recombinase is green in S6/S9 but blue in S8).
