# Supplementary material for: Extensive mobilome heterogeneity in a widespread endosymbiont: A candidate dimeric plasmid and highly fragmented prophages in Wolbachia from Culex pipiens
Source: PLoS Genet. 2026 Oct 26;22(10):e1012347. doi: 10.1371/journal.pgen.1012347 (PMC13734603; doi:10.1371/journal.pgen.1012347)
Supplement: Supplementary file 8 [file pgen.1012347.s018.pdf]

Overall assessment

I thank the authors for their thoughtful responses to my comments, questions, and suggestions. I continue to find this an interesting and potentially valuable study, with a useful resource, interesting techniques, and thoughtful analysis. Many of my previous comments have been addressed. Two main issues remain: the robustness of the assembly and the strength of the biological claims about pWCP.

Assembly robustness

I thank the authors for their additional assembly comparisons and documentation in the notebook. I appreciate the authors' concern that restricting the input to only longer reads could reduce coverage and low-abundance sequence diversity, but I did not see any report or comparison of the longer-reads-only assembly approach.

I don't mean to be a stickler, but the rationale for the importance of testing a longer-reads-only subset (and probably discussing this explicitly in the Methods), especially for this system, is that longer read distributions are more likely to resolve repeats, improve contiguity, and improve assembly quality when read N50 is lower, even though this can compromise small plasmid recovery (e.g. Wick et al. 2023). <u>Thus, the authors' concerns are indeed highly valid, and I am not necessarily suggesting that a longer-read-only assembly should replace the main assembly in the subsequent analyses or recover every rare WO configuration</u>; rather, it is a sensitivity test of whether the core and pWCP structures remain stable. This matters because mishandling of repeats and erroneous duplication of circular plasmids are common mistakes assemblers can make, especially Flye. As assembly is only a consensus, the subsequent raw read analyses are the right place to examine structural heterogeneity and remain valid regardless.

I suggest that the authors include this assembly comparison in the Methods. Another/additional option would be combining several reasonable assembly strategies (e.g. using Autocycler to harmonize Flye/Flye-long-only/Canu/etc. outputs), which might produce the highest quality consensus and also recover the circular plasmid. I think this would strengthen confidence in the assembly as a resource for the scientific community.

pWCP structure and biological interpretation

I appreciate the added read coordinates, adapter check, more cautious treatment of shorter reads, and moderated replication language. I also agree that circular-plasmid reads will have variable endpoints, and that a scarless tandem would not have a unique biological breakpoint. However, this does not answer my structural question. Please show whether the approximately 18.5-kb sequence consists of two simple head-to-tail pWCP units (I think this is what the authors are saying here) and whether their junction differs from an expected tandem duplication.

The assembly and the four long reads make a biological multimer plausible, but they do not establish it uniquely. The host/lambda filter is useful for chimeras involving different source genomes, but it cannot remove a pWCP-pWCP ligation product or other same-reference artifact. Likewise, the absence of an internal adapter is reassuring but does not exclude

every *in-silico* concatemer or “foldbacks” (a.k.a. duplicated inversions). ONT ligation libraries can generate chimeric molecules and substantially underrepresent small plasmids (Wick et al. 2021), while foldbacks and other long-read artifacts can lack detectable internal adapters, map with high confidence, and recur in multiple reads (Heinz et al. 2026). I’m not really an expert on this (and some of these artifacts could be explained or discounted) but I think these artifacts may be common enough that they should be considered and discussed in this context, especially due to the required read filtering.

The result could alternatively be described as tandem or multimeric pWCP sequence consistent with a stable biological multimer, a rolling-circle replication intermediate or concatemer, a recombination product, or a technical artifact. Without stronger evidence, the data do not establish a stable discrete dimer, monomer/dimer coexistence, active replication, or a functional oriV. The categorical wording in the Title and Conclusion should perhaps therefore be moderated.

Minor remaining comments

I appreciate the additional analysis of possible WO connections, and am not requesting further analysis. However, I find the result still a bit difficult to follow. It would be helpful to have a visual representation or clearer description & quantitation of the read-level connections between contig 2 and WO regions, and whether any reads support a given chromosomal region without WO or with different WO integrations / structural arrangements.

Finally, please check the contig counts in the Results: 24 initial contigs minus 10 excluded contigs appears to give 14 retained contigs, consistent with one core contig, one plasmid contig, and 12 smaller WO-rich contigs, rather than 15 retained contigs and 13 WO-rich contigs. But I may have just miscounted this.
