# Supplementary material for: Extensive mobilome heterogeneity in a widespread endosymbiont: A candidate dimeric plasmid and highly fragmented prophages in Wolbachia from Culex pipiens
Source: PLoS Genet. 2026 Oct 26;22(10):e1012347. doi: 10.1371/journal.pgen.1012347 (PMC13734603; doi:10.1371/journal.pgen.1012347)
Supplement: Supplementary file 9 [file pgen.1012347.s017.docx]

**Julie Reveillaud**

MIVEGEC (Univ. Montpellier, INRAe, CNRS, IRD)
IRD, 911, Avenue Agropolis
BP 64501
34 394 Montpellier Cedex 5 – France

reveillaud.j@gmail.com

June 12^th^, 2026

Dear *PLOS Genetics* Editorial Team,

I thank you for your consideration of the manuscript “**Extensive mobilome heterogeneity in a widespread endosymbiont: dimeric plasmids and highly fragmented prophages in *Wolbachia* from *Culex pipiens*** ". The constructive criticism and suggestions of the two reviewers are greatly appreciated and have helped to make the paper stronger. We have taken into account all the reviewer's concerns and comments in the revised version of the paper, and provide point-by-point responses to all comments in the answer to reviewers.

We hope that you will find the revised version of the manuscript suitable for publication in *PLOS Genetics*.

All the authors agree with the new submission of the manuscript, and it is not under consideration elsewhere.

We thank you again for handling our study and for your guidance.

Sincerely,

On behalf of all authors,

Julie Reveillaud

**Point by Point response to Reviewers**

**Reviewer #1:**

This manuscript presents a technically innovative study of the *Wolbachia* mobilome in *Culex pipiens*, using long-read sequencing of a single ovary without prior DNA amplification. The approach is well motivated and addresses an important limitation in the field, namely the difficulty of resolving repeat-rich mobile genetic elements (MGEs), especially prophage WO regions and plasmids. The study provides three main contributions: (i) a near-complete Wolbachia genome reconstructed from a single tissue, (ii) evidence for both monomeric and dimeric forms of the pWCP plasmid, and (iii) read-level observations suggesting structural complexity in WO prophage regions.
However, in its current form, I find that the manuscript is primarily methodological, and the biological insights remain limited or insufficiently developed. While the dataset is valuable, several conclusions are either overstated or not clearly connected to broader biological questions relevant to Wolbachia evolution, host interaction, or vector control.

Specific comments:

The manuscript convincingly demonstrates technical feasibility but provides limited new biological insight. The authors should better articulate how their findings advance our understanding of *Wolbachia* biology (e.g., implications for host interaction, cytoplasmic incompatibility, or genome evolution), beyond documenting structural variation.

We thank the reviewer for his insightful comment. We agreed that the biological implications of our findings were not sufficiently emphasized in the original version of the manuscript. Accordingly, we added a new discussion paragraph to better contextualize how the structural heterogeneity observed within the *Wolbachia* mobilome may contribute to our understanding of *Wolbachia* biology, particularly regarding host interaction, cytoplasmic incompatibility-associated regions, prophage evolution, and genome diversification processes (new lines 358-368 and 470-480).

The data support the presence of dimeric plasmid forms but do not directly demonstrate replication or functional activity. These claims should be reframed more cautiously.

We acknowledge the comment of the reviewer. We agree that the current data do not directly demonstrate active replication or functional activity of the plasmids. Accordingly, we made sure those claims are only suggested in the discussion section, and reworded claims in the results section that were too interpretative. We also removed the word « dynamics » from the title since our presented data is insufficient to prove dynamics or activity, and to better reflect the main findings of the article.

In its current form, the manuscript provides limited support for structural heterogeneity. Several proposed WO/cif configurations are supported by few reads, making conclusions about within-host diversity tentative.

We agree that several proposed WO/*cif* configurations are supported by only a limited number of long reads, which warrants caution in the interpretation of within-host structural heterogeneity. However, even a small number of supporting reads may represent substantial sampling of the bacterial/prophage population in this “host-associated” context. Reads originate from an intracellular symbiont residing within the cells of a eukaryotic host whose genome is considerably larger. The Wolbachia genome is approximately 1.3 Mb, whereas the Culex genome is approximately 580 Mb—nearly 400 times larger. Metagenomic sequencing of host-associated microbiomes faces a fundamental challenge: host DNA can overwhelmingly dominate sequencing libraries, reducing the representation of bacterial DNA and complicating both community profiling and genome reconstruction. In this context, the recovery of multiple long reads (up to 9 long reads > 10kb, out of ca 680 for *Wolbachia*, please see new Table S2,) supporting alternative WO/*Cif* configurations is noteworthy, although we agree that additional evidence would strengthen these observations. In additional, structural heterogeneity of phages is supported by the overall high fragmentation of the assembly. We made sure to highlight this fragmentation in our claims of structural heterogeneity in the results and discussion section.

Despite improved methods, key regions (notably WO) remain unresolved. The manuscript should more clearly acknowledge these limitations and avoid overstating completeness.

We agree that, despite the methodological improvements presented in this study, several WO-associated regions remain only partially resolved due to their highly repetitive nature and structural complexity. Accordingly, we added a sentence in the Discussion section (current lines 365–368 and 419-422) to more explicitly acknowledge these limitations and moderate the interpretation of genome contiguity obtained with the present approach.

The manuscript does not provide strong evidence for phage activity or precise copy number estimates. Conclusions about phage dynamics and gene copy number rely on indirect measures and should be presented with appropriate caution.

We thank the reviewer for his important remark. We agree that the current data do not provide sufficient evidence regarding phage activity and that copy number estimations as observed by qPCR and linear regression analysis should be presented cautiously. Accordingly, we revised the manuscript throughout to use more cautious and appropriate wording when discussing inferred phage copy number, such as in caption 425. We also removed the word « dynamics » from the title to better reflect the main findings of the article.

It is unclear how representative a single ovary is of the broader strain or population.

We acknowledge the reviewer’s comment that our observations are based on a single ovary. However, previous work indicates substantial variability and rearrangement patterns among strains and populations. We compared our results with those of recent studies that used long-read sequencing to investigate similar patterns in *Culex* strains, in line with our results (although these studies did not consider potential PCR-related biases) ; please see (lines 434–442):

“Recent long read studies across *w*Pip groups have likewise reported distinct syntenies around *cif* genes [56]. In the *w*Pip-I (Tunis strain), three different *cif* adjacent environments were documented and termed *cif*-Type I genomic islands. Consistent with these findings, inspection of the raw long reads from *w*PipCelG (*w*Pip-I) also identified distinct *cif*-Type I genomic islands. While the overall pattern agrees with the *w*Pip-I (Tunis) report [56], the exact compositions are not identical: one additional configuration observed in *w*PipCelG (Palindrome 2) was not previously reported, whereas one of the previous configurations (Tunisp-d) is absent from our dataset, suggesting that variability in these regions is likely not dependent on *w*Pip group”.

In a previous study from the group, we also observed SNPs within core gene of *Wolbachia* genomes in distinct *Culex pipiens* ovary samples, highlighting inter-individual variability (Trouche et al., 2024). For this reason, we added a statement in the Methods section (lines 547- 550) clarifying that sequencing a single ovary not only avoids potential amplification-related artefacts but also preserves the genomic architecture of an individual *Wolbachia* population, thereby avoiding the pooling of potentially distinct variants from multiple mosquitoes.

Overall, we believe the data support consequent structural variability at the broader strain and population level that warrant further investigation with a higher number of samples at both the core and accessory genome.

Overall, the study presents a useful technical advance and an interesting dataset. However, the manuscript would benefit from a clearer and more restrained interpretation of the data, as well as a stronger emphasis on biological relevance.

We thank the reviewer for their time, constructive comments, and detailed feedback.

**Reviewer #2:**

**Overall assessment**

This is an interesting, thoughtful, and potentially valuable study. The amplification-free,

single-ovary ONT strategy is well matched to the biological question, and the manuscript is generally clear, well organized, and thoughtfully analyzed. In particular, the careful inspection of raw reads, the caution around assembly limitations, and the well-documented computational notebooks are appreciated. The study provides a useful high-contiguity *w*Pip genome, a nice proof of concept for applying long-read sequencing to low-biomass, host-rich Wolbachia samples, and some intriguing new evidence for *w*Pip’s potential mobilome dynamics; overall it lays useful groundwork for future studies to build more knowledge in this exciting area.

I have several recommendations that I hope will help strengthen the manuscript, and that I hope the authors will find helpful. First, the study would benefit from clearer reporting of the sequencing, filtering, assembly, and contig-curation decisions, since these choices are central to interpreting the results. Second, while the manuscript is valuable as-is, some small additional analysis and contextualization would make the broader claims about mobilome dynamics and MGE activity more convincing.

**Major comments**

**Sequencing strategy, carrier DNA, possible artifacts**

The sequencing approach (single ovary, amplification-free, lambda carrier spike-in) is interesting and seems to make sense. As it is uncommon/novel and central to the manuscript, the decisions should be justified and discussed more explicitly. For example, the rationale for avoiding PCR is reasonable, but the manuscript would benefit from a clearer explanation of the tradeoffs involved, including advantages of avoiding amplification, possible biases introduced by carrier DNA, and placing these approach decisions in context of the recent literature (e.g. other low-biomass microbiome study approaches or precedent for using carrier DNA).

We thank the reviewer for his helpful comment and for pointing us toward the recent literature on low-biomass sequencing approaches. We agree that the rationale and tradeoffs associated with our sequencing strategy deserved further clarification. Accordingly, we revised the “Long read sequencing” section of the Methods to better justify the use of a single-ovary, amplification-free approach combined with lambda carrier DNA, lines 556-566.

A more complete summary of read filtering and read assignment would also improve confidence in the downstream analyses. In particular, it would be helpful to report more stats e.g. reads/bases assigned to host/lambda (& filtered out), *Wolbachia* chromosomal regions, plasmid regions, WO-rich contigs, low-confidence/multimapping categories, and unassigned categories. This is especially important because some central conclusions depend on relatively small numbers of informative long reads. Maybe a flowchart schematic would be helpful.

We thank the reviewer for his insightful suggestion. Regarding the filtering workflow and read assignment, we provide a new supplementary table (S2 Table) summarizing the main sequencing and filtering statistics across the different categories of reads. This table reports the number of reads, number of reads >10kb, total bases, mean read length, mean read quality, N50 values, longest read length, and percentage of total reads for: (i) raw reads passing Guppy quality filtering, (ii) host-assigned (*Culex*) reads, (iii) lambda phage reads, (iv) host metagenome reads retained after host/lambda filtering, (v) *Wolbachia* reads, (vi) secondary/multimapping *Wolbachia* reads with low MAPQ values, and (vii) unassigned host metagenome reads. We agree that these additional statistics are important given that some conclusions rely on a limited number of highly informative long reads, and we hope this additional table improves the clarity and robustness of the sequencing workflow and downstream analyses.

The direct analysis of raw long reads nicely avoids potential assembly artifacts. However, ligation-based ONT libraries can still generate chimeric molecules (the authors mention this briefly in the Discussion; some evidence it can be ~1%). The current adapter-checking step is useful, but the manuscript should more directly address whether host/lambda/*Wolbachia* split-read artifacts could affect filtering or interpretation. For example, during the filtering step, reads could be screened/classified for split alignments, rather than just their best-hit taxonomic assignment, to see whether this is an issue. I don’t know if this will turn anything up and don’t expect it to affect your conclusions, but would be a good due diligence to increase confidence in the results, since they rely on direct inspection of a small number of raw reads.

We thank the reviewer for this thoughtful and very relevant comment. We agree that potential chimeric reads represent an important consideration in ligation-based ONT sequencing approaches. However, in our workflow, potential host/lambda–*Wolbachia* chimeric reads were effectively excluded during the filtering process (see Notebook 1). Importantly, filtering was not performed using taxonomic best-hit assignment approaches (e.g. BLAST), but through direct read mapping against the *Culex* host genome and the lambda genome using **minimap2**. Consequently, any read containing even a small region mapping to either the host or lambda reference was removed from the filtered dataset. This conservative strategy therefore excludes potential chimeric reads involving *Wolbachia* and either the mosquito host or the lambda carrier DNA.

In addition, all manually inspected reads used for structural analyses were individually annotated and carefully examined, and we did not identify any obvious chimeric read among them. Similarly, we did not detect residual adapter sequences that could indicate ligation-mediated chimeric artifacts.

[154] Accordingly, please soften the claim that the sequencing strategy avoids chimeric sequences. Avoiding PCR reduces one source of chimerism, but ligation-based longread sequencing can still generate chimeric reads. The later discussion around 428–434 is more cautious and could be made consistent with the earlier wording.

We thank the reviewer for his comment. We agree that the original wording is too categorical regarding the avoidance of chimeric sequences. Accordingly, we revised the sentence (current line 146) to adopt a more cautious formulation, clarifying that the absence of PCR amplification was intended to limit amplification-associated biases and potential PCR-induced chimeric sequences.

“We developed an *in-house* protocol leveraging the sequencing capacity of MinION technology (ONT) without any upstream nucleic acid amplification in order to reduce the introduction of possible PCR errors and chimeric sequences between highly repeated genomic regions.”

[Methods] Please cite or briefly discuss precedent and tradeoffs for lambda carrier DNA in low-input sequencing; state the flow cell details and pore chemistry; clarify how atypical contigs likely occurred.

We revised the “Long read sequencing” section of the Methods to provide additional details regarding the sequencing setup, including the flow cell and pore chemistry used for the Nanopore run as suggested. We also expanded the description and discussion of the lambda carrier DNA strategy in the context of low-input sequencing approaches (lines 556 – 566 and 570).

**Assembly parameters and contig curation**

The author’s approach of manual contig curation and avoiding over-interpretation of the assembly are generally reasonable, but the manuscript should provide more detail about the origin of the excluded contigs and the robustness of the final assembly. The contigs with negligible coverage or 100% identity to internal regions of larger contigs (line 202-209) are somewhat surprising to me, especially for recent versions of Flye (unlike e.g. older versions of CANU where I might expect some of this more).

We thank the reviewer for this relevant comment. Regarding contigs with negligible coverage, unsupported or potentially artifactual contigs have previously been reported even with recent versions of Flye. For example, a recent study published by Trigodet and colleagues (2026) in [Nature Biotechnology](https://doi.org/10.1038/s41587-025-02971-8) (doi: https://doi.org/10.1038/s41587-025-02971-8) (Fig. 2) reported contigs lacking read support despite the use of recent Flye assemblies, suggesting that such low-support or potentially artifactual contigs may still occasionally occur in long-read assemblies.

As for contigs with 100 % identity, we believe this is a consequence of the larger WO phage reads being unresolved in their structural heterogeneity, as shown in Fig. 5 of the current manuscript. To improve clarity about manual contig curation, we revised S4 Table to provide detailed characteristics for each contig identified in the assembly, including coverage information and explicit indication of contigs excluded from downstream analyses, together with the reason for their exclusion (absence of coverage support or complete identity with larger contigs).

It would be useful to provide more information for accounting of these contigs — whether they are low-support contigs, multimapping/repeat-derived contigs, residual host-like sequence (e.g. related to *Culex* masking choices and EAM phage regions), or artifacts related to assembly and filtering choices (detailed below).

As described above, reads potentially containing host/*Wolbachia* or lambda/*Wolbachia* chimeric regions were removed during the filtering step through direct mapping against the *Culex* and lambda reference genomes rather than taxonomic best-hit assignment approaches. Consequently, reads containing even small host- or lambda-derived regions were excluded from the filtered dataset. Because our objective was to perform a fully *de novo* assembly, the assembly was initially generated using the complete filtered read dataset. We subsequently evaluated contig support by remapping reads onto the assembly using primary alignments with MAPQ > 2 in order to limit ambiguities related to multimapping or repetitive regions. Contigs lacking sufficient primary mapping support were therefore excluded from downstream analyses. To improve clarity about manual contig curation, new S4 Table was yet also updated to provide additional details regarding contig characteristics, coverage support, and the rationale underlying contig exclusion.

Because the highly contiguous *w*Pip genome is one of the study’s main outputs, the assembly parameters should be reported directly in the Methods, including Flye version and key options. It would also be helpful to briefly state whether alternative common assembly settings were considered and whether they affected the main chromosome, plasmid, or WOrich contigs. If the authors have not already considered, I suggest several specific recommendations here:

1. Run Flye filtered only to longer reads (maybe >2 kb), and include the —keep-haplotypes flag. This may help improve assembly contiguity and the ability to resolve the possible phage structural variants.

2. In general the —nano-raw flag is only recommended for older technology (higher error rate and older Guppy). Referring to the Flye usage docs on GitHub, using —nano-hq for Guppy5+ SUP and adding —read-error .05 seems to be the recommended choice based on the data here.

3. The —meta mode was probably the correct choice (as noted by the authors in the workbook), but it might be worth running Flye without it to see if this improves chromosome or plasmid contiguity at all. The existing parameter choices may already be optimal for this particular dataset, but the

authors should consider, test, and justify their parameter choices, given that the resulting genome is a key contribution of the study.

We thank the reviewer for these very helpful suggestions. Following this comment, we expanded the Methods section to explicitly report the Flye version and assembly parameters used in the study (current line 583-586 of the manuscript). In addition, we added a new supplementary notebook (S2 Notebook) presenting the different assembly strategies tested, including new assembly attempts using the parameters recommended by the reviewer (`--keep-haplotypes`, `--nano-hq` and `--read-error 0.05`) (S2 Notebook). Comparison of these assemblies indicates that the assembly retained in the present study remains among the most relevant in terms of overall contiguity and number of recovered contigs for this specific dataset. Importantly, one of the main conclusions of this work is that conventional assembly approaches remain unable to fully resolve highly repetitive WO-rich regions and may generate fragmented or potentially chimeric structures, particularly within prophage-associated loci. Consequently, much of our interpretation ultimately relies on the direct analysis of raw long reads rather than on assembly structure alone.

We also tested the assembly configurations restricted to reads >2 kb and combined with the `--keep-haplotypes’ option. However, we were concerned that restricting the dataset to longer reads only could remove a substantial fraction of the available sequence diversity and reduce coverage across low-abundance genomic regions. Notably, restricting to a subset of data by removing reads of size under 2 kb, could get rid of important information such as for *cif* regions for which reads are very fragmented. Overall, while these approaches reduced the total number of contigs, notably by removing contigs supported only by short reads, they did not resolve the main challenges associated with WO-rich regions. Indeed, despite the use of the `--keep-haplotypes’ flag, these assemblies still produced fragmented structures and highly heterogeneous coverage profiles along several WO-associated contigs, as illustrated in the figure below showing the alignment of reads to a >70 kb contig containing duplicated *cif* regions.

Overall, we greatly appreciate these suggestions, which allowed us to more thoroughly explore and document the behavior of different assembly strategies on this challenging low-input and repeat-rich dataset.

**pWCP plasmid dimerization & replication evidence**

The pWCP results are compelling, but the interpretation should more clearly separate observed structures from proposed mechanisms. The evidence for monomeric and dimeric/multimeric pWCP forms should be presented separately from the evidence for active replication. A dimeric plasmid, a replication intermediate, an unresolved recombination product, and a concatemer are related but distinct possibilities. The manuscript discusses these possibilities, but the Results and Discussion would be clearer if the various observed read/contig structures were distinguished more explicitly from each other and from the mechanistic interpretation.

We thank the reviewer for the thoughtful comment. We agree that the distinction between directly observed plasmid structures and the mechanistic interpretations associated with them needs clarification. Accordingly, we revised the Results and Discussion to better distinguish evidence supporting monomeric and multimeric/dimeric pWCP configurations from the more speculative hypotheses related to plasmid replication mechanisms. In particular, we reorganized the presentation of the different observed read structures and more clearly separated the description of the observed plasmid conformations from the discussion of possible underlying mechanisms, including replication intermediates, concatemer formation, or unresolved recombination products. We also moderated several formulations throughout these sections to more cautiously present the proposed replication-associated interpretations, such as lines 43-44, 246 or 372-373.

It would also help to clarify how the 9 kb and 18 kb forms relate to one another structurally, especially around the proposed oriV/VNTR region and what the inferred dimer junction looks like (is the 18 kb form a “fused plasmid” via IS/transposon-mediated recombination? Does the 9kb form split near the oriV? etc.)

We thank the reviewer for this insightful comment. Because pWCP is a circular plasmid and Nanopore reads are linearized randomly during sequencing, reads do not systematically start or end at the same position along the plasmid sequence. Accordingly, the observed long reads supporting monomeric or multimeric configurations exhibit heterogeneous start and end coordinates rather than a consistent breakpoint or junction region. We have therefore added a new supplementary table (S5 Table) reporting the start and end coordinates of all analyzed plasmid reads, BLASTed against the oriV/VNTR region (corresponding to positions 6,363 – 6,663 on contig_9), illustrating this random distribution across the plasmid sequence. No junction structure is observed; indeed the observed plasmid sequences seem independent of the VNTR region, and the IS or other DNA was not observed to ligate plasmid fragments in any way. Consequently, we cannot confidently infer a unique or recurrent “fusion” mechanism between the 9 kb and 18 kb forms, nor determine whether multimeric configurations specifically form near the proposed oriV/VNTR region.

For example, on line 239-240, please clarify whether the shorter reads uniquely support the 9 kb monomeric form or are also consistent with reads sampled from larger circular or multimeric structures but are just not long enough to know. If the 18 kb form differs from the 9kb form near the putative oriV or at other junctions, it would be interesting and helpful information to provide.

We thank the reviewer for his relevant comment. We agree that shorter plasmid reads do not necessarily uniquely support the canonical 9 kb monomeric configuration. Indeed, some intermediate-length reads (for example >10 kb) may also be compatible with larger multimeric plasmid structures but remain too short to unambiguously resolve the complete plasmid configuration. We clarified this point in the revised manuscript (lines 234–236). In addition, these reads (>10 kb) could also support an interrupted replication mechanism as shown in Fig S4, and as discussed in the Discussion dimers could be the result of an interrupted replication mechanism.

Reads start and end at heterogeneous positions along the plasmid sequence rather than at a conserved breakpoint. Overall, data do not support a specific junction structure/hypothesis. To clarify this point, we added a new supplementary table (S5 Table) reporting the alignment coordinates of all analyzed plasmid reads on the OriV/VNTR region, illustrating this random distribution across the plasmid. We also refer to Supplementary Notebook 5 for additional information.

**WO phage integration, structural heterogeneity, potential activity**

The WO phage results are exciting, but the interpretation would be stronger with more direct read support for integration sites and structural relationships among WO-rich contigs. The current data support substantial under-assembled WO diversity, but several models remain possible: multiple chromosomal prophage loci, tandem structures, relic fragments, episomal or lytic phage, or rare within-host structural variants (though I’m not very familiar with what has already been specifically established about WO phage biology/genetics). The manuscript would benefit from a clearer distinction among these possibilities and, where possible, stronger read-level evidence linking WO-rich contigs to their potential chromosomal context. I suggest some small additional read-level analysis which could provide more compelling evidence for phage dynamics:

We thank the reviewer for this thoughtful comment and fully agree that the structural organization and biological nature of the WO-associated regions identified in this study require careful interpretation. As pointed out by the reviewer, several alternative models may explain the observed patterns, including multiple integrated prophage loci, tandem prophage arrangements, relic phage fragments, episomal or lytic WO forms, and potentially rare structural variants coexisting within the same host. Given the complexity of these repetitive regions and the limitations of the current assembly, we agree that distinguishing among these possibilities is essential.

In response to these comments we address each of the reviewer's suggestions point-by-point below.

• [296] “observed core genes flanking EAM regions in almost all contigs” — where are these core genes located on contig_2? This could provide evidence for the putative phage integration site(s).

We thank the reviewer for the comment. Here, the term “core genes” refer specifically to conserved WO phage core genes flanking EAM regions, as defined in a previous comparative genomic study based on an exhaustive analysis of WO prophages in *Wolbachia* (doi: 10.1371/journal.pgen.1010227). The positions of these phage-associated genes within contig_2 are already indicated slightly earlier in the manuscript (current lines 281–287). To further improve clarity and provide additional contextual information regarding neighboring genes, we also added the complete GFF annotation file together with Pfam annotations in S6 Table.

• [301/315] The authors note that the *cif* and Octomom-like sequence at contig_2 edges strongly suggest a phage-associated integration region. More explicit read-level support would strengthen this interpretation: are there any reads mapping to the ends of contig 2 which span across this putative phage integration site? And/or do the ends of any phage contigs (especially the ones with more uniform coverage) map back to contig_2? This could also be done at an individual read level. The Flye —keep-haplotypes flag (or running a tool like Sniffles2) could also be helpful here.

We thank the reviewer for this insightful suggestion. Indeed, several long reads mapping to the terminal region of contig_2 also partially map to contig_10 and contig_22 (see IGV screenshot below, red rectangle), supporting the hypothesis that these regions likely correspond to prophage integration-associated regions. However, this point remains at the contig level. We did not recover any sufficiently individual long reads spanning both clearly identifiable *Wolbachia* chromosomal core genes within contig_2 and extended prophage regions containing canonical WO phage structural genes outside the EAM module. Consequently, although the observed read mappings support the presence of phage-associated regions connected to contig_2, the available data do not allow confident resolution of the exact structural organization or integration state of these prophage regions. We added these clarifications to the revised manuscript in order to strengthen and better contextualize the interpretation regarding putative prophage integration sites (current lines 290-295).

• [309] "These values suggest that the genome contains multiple intact and relic prophage

elements…" — I find this to be an exciting result that’s worth digging into a bit more.

- Can the authors provide confident evidence that the sr3WO LSRs on contig_3 and contig_10 are genuinely different (rather than reflecting ONT error, collapsed repeats, or assembly ambiguity)? If so, this provides another piece of evidence to support the claim that the genome contains multiple prophage elements.

The sr3WO large serine recombinase (LSR) sequences identified on contig_3 were extracted from their respective contigs and compared at the gene sequence level. The observed sequence differences occur within the coding regions of the sr3WO genes, including the terminal portion of the gene itself (as illustrated below), supporting the idea that these sequences are unlikely to simply result from ONT sequencing errors, collapsed repeats, or assembly ambiguities. However, these remain within the same sr3WO clade. Indeed, the presence of multiple WO prophage elements in *Wolbachia* from *Culex* mosquitoes has already been reported in previous studies (Klasson et al., 2008; Bordenstein & Bordenstein, 2022). Therefore, our observations are consistent with the known complexity of WO prophage organization in *w*Pip genomes and mainly suggest that the number and structural diversity of these prophage-associated regions may be more variable than previously appreciated.

- If there are multiple prophage elements, they are either in tandem or they have multiple integration sites in the chromosome. Furthermore, for a given chromosome, if there is read support for any chromosomal loci both with and without WO region integrated, this would heavily suggest phage activity.

We thank the reviewer for this relevant point and suggestion. As described above, we specifically investigated these possibilities by examining individual reads potentially linking prophage-associated regions to chromosomal regions. However, we did not recover sufficiently long reads simultaneously spanning clearly identifiable WO prophage structural regions (i.e. containing canonical WO core phage genes beyond the EAM module) together with confidently assigned chromosomal regions.

For example, reads mapping to both contig_2 and contig_10 predominantly contained either EAM-associated phage regions only (including Octomom-like and *cif*-associated regions) or EAM-associated regions together with nearby chromosomal genes located at the extremities of contig_2 (see GFF annotations of contig_2, S6 Table). Consequently, although these observations support structural associations between contig_2 and several WO-rich contigs, the current dataset does not allow us to confidently determine whether these prophage regions correspond to multiple chromosomal integration sites, or tandem structural configurations. Furthermore, even if reads spanning both canonical WO structural genes and adjacent *Wolbachia* chromosomal regions had been recovered, their interpretation would not necessarily be straightforward. Phage-mediated DNA packaging can result from distinct mechanisms, including specialized transduction, in which bacterial sequences adjacent to prophage integration sites are co-packaged together with phage DNA. Therefore, the detection of reads linking WO core phage regions and neighboring chromosomal loci would not, by itself, allow us to distinguish between chromosomal integration patterns, tandem prophage structures, or transduction-mediated packaging events.

Either way, please clarify whether the evidence supports multiple loci, tandem organization, episomal/lytic phage, or some combination.

Based on the current dataset, several structural models remain plausible. We identified individual reads linking EAM-associated regions with chromosomal regions, as well as other reads linking EAM-associated regions with canonical WO core phage genes. However, we did not recover reads directly connecting clearly identifiable WO core phage regions with confidently assigned chromosomal regions. Consequently, the current data remain compatible with several non-exclusive hypotheses, including multiple prophage loci with tandem prophage organization, episomal forms, or lytic phage particles. In addition, recent work has reported evidence for the presence of lytic WO phage particles in *w*Pip *Wolbachia* (doi:10.21203/rs.3.rs-9836465/v1), further supporting the plausibility of such configurations in *Culex*. We therefore agree that clarifying the most likely structural model would be highly valuable. However, given the absence of sufficiently informative long reads spanning complete prophage/chromosomal junctions we do not believe the present dataset allows us to confidently discriminate between prophage integration models and potentially active lytic or episomal phage configurations. We moderated the corresponding interpretations in the revised manuscript (current lines 419-422).

[395] ”highly repetitive nature of prophage regions and possibly the presence of active phage particles”. Please distinguish evidence for assembly fragmentation from evidence for active phage particles; more light could be shed on this by looking at read ends.

As discussed above, examination of read extremities unfortunately did not provide sufficient additional information to confidently discriminate between assembly fragmentation associated with repetitive prophage regions and the potential presence of active phage particles. We nevertheless clarified this limitation in the revised Discussion and added a sentence explicitly acknowledging this point (lines 419-422).

From the existing results presented in this section, I’m not convinced that the authors have provided enough evidence to claim “active” phage “dynamics” here. If the authors feel that any additional analyses would be out of scope for this manuscript, that’s okay, but in that case, I think they should soften their claims about phage dynamics a bit. The existing results are intriguing and consistent with phage activity, but also potentially consistent with other possibilities. It’s possible that some small additional read-level analysis would provide additional evidence for phage dynamics.

Again, we thank the reviewer for his thoughtful comments related to this matter. As discussed above, we thoroughly explored several additional read-level analyses to better distinguish between the different possible WO phage configurations and to search for stronger evidence supporting active phage dynamics. However, the current dataset did not provide sufficiently informative reads to confidently discriminate between the different structural scenarios discussed above. We therefore agree that, given these limitations, usage of the term “dynamic” in the title and some parts of the manuscript is inadequate. Accordingly, we revised and moderated our wording throughout the manuscript, including the title, Results, Discussion, and Conclusion sections, to better reflect that the observed patterns are consistent with structural heterogeneity and possible phage activity, while remaining compatible with alternative interpretations.

**Inference of MGE activity and mobilome dynamics**

The framing of an active, dynamic *Wolbachia* mobilome is plausible, but

[Title] I’m admittedly a bit hesitant about the word “dynamics” here, though it’s reasonable if the manuscript makes clear that some inferred dynamics come from structural heterogeneity rather than direct temporal observation.

We thank the reviewer for this thoughtful remark and agree that the term “dynamics” could imply direct temporal observations or experimentally demonstrated activity, which is beyond the scope of the current dataset. Accordingly, we revised the title to better reflect that the inferred mobilome variability primarily arises from structural heterogeneity observed within the sequencing data.

The new title is now: “Extensive mobilome heterogeneity in *a widespread endosymbiont:* dimeric plasmids and highly fragmented prophages in *Wolbachia* from *Culex pipiens*”.

[352] More cautious wording such as “putatively active,” “structurally heterogeneous,” or

“consistent with activity” may be more appropriate depending on the specific claim. I’m not strongly against “active MGE” but would carefully consider the conclusions supported by the data.

We agree with the reviewer’s comment. We carefully revised the manuscript and moderated several formulations throughout the text in order to better distinguish direct observations from mechanistic interpretations. In particular, terms such as “active,” “dynamic,” or related formulations were replaced or nuanced where appropriate using more cautious wording depending on the context and the level of evidence provided by the data.

The interpretation of within-host structural heterogeneity would benefit from a clearer discussion of possible *Wolbachia* strain heterogeneity within a single organ. I don’t have domain knowledge here, but my understanding is that although within-host *Wolbachia* populations are often comprised of multiple strains, previous work by this group has shown there is remarkably little strain diversity for *w*Pip in *C. pipens* specifically. The authors could even provide evidence in these data for low intra-individual diversity (e.g. Strainy on the existing assembly graph, or InStrain from the short-read data, etc.).

This would increase support for the authors’ claim that what we’re observing here is structural heterogeneity against a single genomic background, rather than strain heterogeneity.

We agree that discussing structural heterogeneity vs strain heterogeneity is an important point. At present, we are actively investigating these working hypotheses, but it remains technically challenging to resolve with confidence using the current dataset. The main limitation is that the structural variants observed in our data cannot be unambiguously assigned either to distinct Wolbachia strains or to alternative genomic configurations coexisting within the same bacterial cell or chromosome, as mentioned. Consequently, while the observed patterns could reflect the presence of slightly different Wolbachia populations differing in their mobilome structure, the current data do not allow us to confidently distinguish this scenario from structural heterogeneity occurring within a single genomic background.

We therefore use warranted caution in our interpretation. We thank the reviewer for highlighting tools such as Strainy and inStrain. As mentioned by the reviewer, previous analyses by the team have shown remarkably low levels of within-host genetic diversity on the core genome of *w*Pip in *C. pipiens* spp. However, the ambiguity in our interpretation stems specifically from the accessory genome and mobilome, where the presence of multiple versions of the same element limits confident assignment to a single genomic background. Resolving this question would require haplotype-level phasing, for which tools such as Strainy are more appropriate. For this reason, we are actively investigating its use in future work using ultra-long sequencing reads (>100 kb) and higher coverage data. However, generating such datasets requires substantial protocol optimization for an intracellular symbiont and remains technically demanding, placing it beyond the scope of the current study.

**Minor general comments**

The introduction mentions small MGEs such as insertion sequences and transposons.

Given the nature of the long read data generated here, it might be interesting to briefly

comment on any observed IS/transposon heterogeneity or putative activity in the chromosome (other than what was found on WOPip5).

We thank the reviewer for this suggestion. Quantification analyses of transposase-associated regions across the *Wolbachia* genome were originally presented in Supplementary Figure 8. However, because one of the main conclusions of this study is that assembly reconstruction remains unreliable for several highly repetitive regions, particularly within the mobilome, we primarily focused our interpretation on coverage profiles and direct long-read analyses rather than on assembly-derived structural comparisons alone.

The Discussion could briefly mention how newer ONT chemistry (R10.4.1), basecalling

(Dorado), and adaptive sampling approaches (to preferentially reject host & lambda

molecules) might improve future work on similar low-biomass, host-rich samples.

We thank the reviewer for this suggestion. We added a sentence in Methods, around lines 556-566, commenting on these topics.

“PCR amplification is often used to increase DNA yield when working with low-biomass samples but may introduce amplification biases and alter the relative representation of genomic sequence [59–61]. To minimize these potential artefacts, a direct long read sequencing strategy was employed without prior whole genome amplification. Because the amount of ovarian DNA recovered from a single mosquito was below the recommended input for Nanopore library preparation (1µg genomic DNA), *lambda* phage DNA was added as carrier DNA to reach the minimum required DNA quantity (detailed in **S7** **Table**). Similar carrier DNA approaches have previously been proposed for ultra-low input sequencing applications to improve library preparation efficiency while preserving amplification free workflows [60]. Although carrier DNA may reduce the proportion of informative reads originating from the biological sample, it can subsequently be computationally removed from downstream analyses “.

The fast-evolving long-read sequencing technologies, such as the newest ONT chemistries and basecalling platforms—R10.4.1 flow cells and Dorado basecalling, compared to our current R9.4.1 flow cells and Guppy basecaller—may indeed enable much deeper sequencing of low-biomass samples in the near future, potentially revealing rare phage WO variants and other low-abundance genomic features. However, several challenges and potential disadvantages remain.The extremely large size of the Culex genome compared to *Wolbachia* genomes—a roughly 500-fold difference—poses a substantial obstacle. ONT experts at the London Calling 2022 event suggested that adaptive sampling, while promising for targeted enrichment, may lead to rapid exhaustion of sequencing pores when applied to such disproportionately large host genomes (personal communication to JR). This could result in dramatically reduced output, limiting the recovery of the target low-abundance sequences and this approach was therefore avoided in this context. The newer chemistries and basecallers, while improving raw read accuracy and throughput, can introduce biases in sequence representation, particularly for regions with high GC content, repetitive sequences, or modified bases. Moreover, while the theoretical yield per flow cell is higher, practical issues such as pore clogging, library preparation variability, and DNA degradation in low-biomass samples may still constrain effective sequencing depth.

**Minor line-by-line comments**

[155] The term “punctual mutation” may be more of a French-specific convention; I’d recommend “point mutation ” (or SNV depending on the intended meaning).

We thank the reviewer for pointing this out. The term “punctual mutation” has been replaced by “point mutation”.

[174] “Same strain” appears to refer to the mosquito strain, but this could be confused with Wolbachia strain. Please ensure that the intended meaning is clear (here and several other places in the manuscript).

We revised the wording throughout the manuscript to clearly distinguish the mosquito strain from the *Wolbachia* strain whenever relevant.

[Fig. 1] Consider adding anatomical labels (for readers less familiar, given the journal audience). Relatedly, some additional background on WO phage biology might be helpful for readers unfamiliar with the domain.

We added a sentence about WO phage biology in the introduction “Among these elements, temperate bacteriophage WO or formally WOvirus is the best studied” (line 91). In addition, we revised Figure 1 to include anatomical labels identifying the mosquito body regions and tissues represented in the schematic. Corresponding explanations were also added to the figure legend to improve accessibility for readers who may not be familiar with mosquito anatomy or the biological system studied.

[278] “Unassembled prophage regions” could be misread as unassembled reads. Please clarify whether this refers to fragmented assembly of prophage loci.

We clarified the wording in the revised manuscript to explicitly indicate that this refers to the fragmented assembly of prophage-associated loci rather than to unassembled sequencing reads (current line 276).

[294] Please clarify whether “the 13 contigs containing prophage WO related regions” refers to contig_2 plus the 12 smaller WO-rich contigs.

We revised the sentence in the manuscript to explicitly specify that the 13 contigs correspond to prophage WO-associated regions identified within contig_2 together with the 12 additional smaller WO-rich contigs (current lines 297-298).

[Fig. 2B / S4C] The path of read 7 is difficult to follow, especially because antisense sequence appears represented differently between panels. Please harmonize or explain the visual conventions.

Thank you for this comment. We improved the captions of fig. 2B / S4C to better explain that inferred paths of reads should be read from the most inner circle to the most outer circle, with the triangle arrows as starting points for each layer. This means read 7's path should be understood as first the small antisense part, then, a full turn over the plasmid in sense, then the small sense part. And for another example, read 2 should be understood as starting, then doing a full turn over the dimeric plasmid in sense, and continuing with a smaller part of the dimeric plasmid in sense. We also want to clarify that the presented paths of fig. 2B / S4C are models based on the alignments of reads to the references of the monomeric or dimeric form of the plasmid.

[Fig. 2B] Please check consistency of the pWCP reference length. The text gives 9,228

bp, while Fig. 2 appears to show 9,243 bp.

We are really thankful to the reviewer for his remark. The Fig. 2. indeed displayed 9,243 bp because read paths shown are based on alignments over the contig_9 split in half (pWCP od *w*PipCelG), in order to represent the expected monomeric form of the plasmid. We erroneously displayed this plasmid reference as « pWCP » but this refers to the monomeric pWCP form of Celestin strain, with ONT mutations, and VNTR nucleotide insertions, rather than to the « pWCP reference » of 9,228 bp as published in the 2019 paper of Reveillaud et al. We corrected the plasmid reference name in the current version of the manuscript.

[Fig. S6] — overall highly informative; maybe worth having as a main figure

We thank the reviewer for this suggestion. Following the reviewer’s recommendation, Supplementary Figure S6 has been moved to the main manuscript (now Figure 3), as we agree that it provides important support for the main conclusions of the study.

The red/phage annotation is a bit difficult for me to interpret. Please clarify whether this reflects specific prophage ORF regions, manual assignment of phage-like contigs, or another criterion.

Consider visually separating the small WO-rich contigs from the chromosomal representation in the Circos plot, as their order/position are not confirmed.

Consider calling contig_2 “chromosomal” rather than “core genome”, unless “core genome” is intended in a specific comparative-genomic sense.

Please add numeric labels to the phage contigs in A, corresponding to the numbering in B.

There are two different grey gene shadesin B, but they’re not labeled (is light grey core genes?)

We thank the reviewer for his note. The requested modifications and clarifications were implemented in the revised figure and/or its legend. In particular, we clarified the meaning of the red phage-associated annotations, added numeric labels corresponding to the WO-rich contigs shown in panel B, clarified the grey color categories, and revised the terminology to improve readability and avoid overinterpretation of contig order and positioning.

[Fig. 4A]

• “Read 7” and “read 8” may be confused with plasmid reads 7 and 8 in Fig. 2B. Consider using more consistent read identifiers.

We thank the reviewer for pointing out this source of confusion. We agree that using the same read identifiers in Figures 2 and 5 was potentially misleading. To improve clarity, the reads have been renamed using distinct identifiers according to their context, thereby avoiding any ambiguity between plasmid-associated and phage-associated reads.

• Where do those reads map to on the contigs shown in S6A/B? I understand the conclusion is that these regions are likely rearranged for wPipCel vs wPipPel, but how read 7 and read 8 map onto WOPip1/WOPip5 and how those map onto the corresponding locations in wPipCel and wPipPel genome is not clear to me.

The reads in question map to contig_22 (see IGV screenshot below), with the newly renamed read 12 shown in red and read 13 shown in blue. Interestingly, contig_22 contains the WOphage1–WOphage5 (see S4 Table) junction observed in the raw long reads, indicating that this arrangement was retained during assembly. However, because the assembly of WO-rich regions remains highly uncertain throughout this study, we considered the direct inspection of raw long reads to be a more rigorous and conservative approach for demonstrating the existence of this junction. For this reason, our interpretation primarily relies on read-level evidence, while contig_22 seems consistent with the observed arrangement.

[Fig. 4B] Palindrome 1 is supported by 8 reads, but based on what’s shown, it seems like some of these 8 reads do not span the entire region? Consider showing a diagram (or igv style plot) to clarify, for each of these palindromic regions, exactly where the read support lies across the region.

We thank the reviewer for this comment. Indeed, the reads supporting the different configurations do not necessarily span the entire region shown in new Figure 5B. Rather, they support a given configuration through unambiguous syntenic patterns involving the organization of *cif* genes (tandem or non-tandem arrangements) and the presence or absence of neighboring genes such as *mutL*, *rnhA*, and *intII*. These features allow individual reads to be assigned confidently to one of the proposed configurations, even when they do not cover the entire region.

We agree that a read-level representation showing the exact coverage of each supporting read across these regions could be informative. However, these configurations were identified through manual inspection of the long reads precisely because conventional mapping approaches are difficult to interpret in these regions. Owing to the high sequence similarity shared among the alternative configurations, many reads can map to multiple configurations, which likely contributes to the inability of the assembly process to resolve and represent them separately. For this reason, we chose to focus on manually curated read-supported configurations rather than mapping-based visualizations (like iGV), which we fear could be misleading.

• Not sure if I’m interpreting this correctly, but could read 2 map well (anti-sense) to

palindrome 2 if intII was mobilized? It’s a bit hard to make sense of what might be happening here.

We thank the reviewer for this insightful observation. Indeed, the newly renamed read 15 (previously read 2) could potentially align to the Palindrome 2 configuration if the *intII* gene were present. Interestingly, the genes *mutL*, *intII*, and *rnhA* appear to be key variable elements distinguishing the different *cif*-associated configurations observed in our dataset. Similar patterns were recently described by Amoros et al. (2026), where the presence, absence, and organization of these genes contributed to the definition of distinct *cif* genomic islands.

• I’m generally skeptical of something that has only 1 read supporting it, but I think it’s

fine to show because the authors are transparent about this.

We appreciate the comment. We believe this is an interesting observation/additional *cif* configuration worthwhile to present, and indeed we remain transparent regarding the strength of supporting evidence.

Figures generally: Consider using consistent colors for functional categories across figures (e.g., recombinase is green in S6/S9 but blue in S8).

We thank the reviewer for pointing out this inconsistency. To improve readability and facilitate comparison across figures, we revised the color scheme to ensure that identical functional categories are represented by consistent colors throughout the manuscript.

We are very thankful to Dr. Aaron A Behr for his comprehensive evaluation and critical suggestions, which strengthened our study.
