# Supplementary material for: Extensive mobilome heterogeneity in a widespread endosymbiont: A candidate dimeric plasmid and highly fragmented prophages in Wolbachia from Culex pipiens
Source: PLoS Genet. 2026 Oct 26;22(10):e1012347. doi: 10.1371/journal.pgen.1012347 (PMC13734603; doi:10.1371/journal.pgen.1012347)
Supplement: Supplementary file 10 [file pgen.1012347.s019.docx]

Response to Reviewers – SECOND REVIEW

**Overall assessment**

I thank the authors for their thoughtful responses to my comments, questions, and suggestions. I continue to find this an interesting and potentially valuable study, with a useful resource, interesting techniques, and thoughtful analysis. Many of my previous comments have been addressed. Two main issues remain: the robustness of the assembly and the strength of the biological claims about pWCP.

**Assembly robustness**

I thank the authors for their additional assembly comparisons and documentation in the notebook. I appreciate the authors' concern that restricting the input to only longer reads could reduce coverage and low-abundance sequence diversity, but I did not see any report or comparison of the longer-reads-only assembly approach. I don’t mean to be a stickler, but the rationale for the importance of testing a longer-reads only subset (and probably discussing this explicitly in the Methods), especially for this system, is that longer read distributions are more likely to resolve repeats, improve contiguity, and improve assembly quality when read N50 is lower, even though this can compromise small plasmid recovery (e.g. Wick et al. 2023). Thus, the authors’ concerns are indeed highly valid, and I am not necessarily suggesting that a longer-read-only assembly should replace the main assembly in the subsequent analyses or recover every rare WO configuration; rather, it is a sensitivity test of whether the core and pWCP structures remain stable. This matters because mishandling of repeats and erroneous duplication of circular plasmids are common mistakes assemblers can make, especially Flye. As assembly is only a consensus, the subsequent raw read analyses are the right place to examine structural heterogeneity and remain valid regardless. I suggest that the authors include this assembly comparison in the Methods. Another/ additional option would be combining several reasonable assembly strategies (e.g. using Autocycler to harmonize Flye/Flye-long-only/Canu/etc. outputs), which might produce the highest quality consensus and also recover the circular plasmid. I think this would strengthen confidence in the assembly as a resource for the scientific community.

Thank you for your comment. We advanced this assembly comparison with new Flye assemblies including reads of over 2,000 bp and the ‘–keep-haplotypes’ parameter in Supplementary Notebook 2, as well as in the Methods section of the article (New lines 627- 636) and in the Discussion (New line 446-448). Results confirm that the core genome and pWCP structures remain stable, namely that pWCP assembly is retrieved and dimeric in six out of eight Flye configurations (notably in all four assemblies including reads of over 2,000 bp) while the core genome has an average, expected length of 1,196,852.875 base pairs across all eight Flye configuration.

Because these alternative assembly strategies did not alter the main observations relevant to this study, we retained the original assembly for the downstream analyses presented in the manuscript and used the additional assemblies as sensitivity analyses of assembly robustness. To ensure transparency and facilitate future use of these data, the results and parameters of all alternative assemblies are documented in **Supplementary Notebook 2**, and we additionally provide on Zenodo (10.5281/zenodo.22935164) the >2,000-bp-read assembly with the lowest number of contigs alongside the assembly used in the main article (with options –keep-haplotypes, --nano-hq and --read-error .05).

We also used Autocycler (Wick et al., 2025), but generally obtained worse results (e.g. smaller core genome at around 700k bp, less than five WO regions). The relatively low sequencing depth of the dataset and repeated regions in the *Wolbachia* genome make Autocycler's approach likely less suitable for our data as it is dependent on the quality of the input assemblies. In addition, its assembly refinement resolved our concatemeric plasmid contig into a single copy. Given that four reads were identified spanning the full length of the putative dimeric plasmid and that a linear replicon removal is mentioned in Autocycler's wiki, this could raise further concerns about its performance on the current dataset and Autocycler's results were not further analyzed here.

We thank again the reviewer for those thoughtful comments and suggestions.

**pWCP structure and biological interpretation**

I appreciate the added read coordinates, adapter check, more cautious treatment of shorter reads, and moderated replication language. I also agree that circular-plasmid reads will have variable endpoints, and that a scarless tandem would not have a unique biological breakpoint. However, this does not answer my structural question. Please show whether the approximately 18.5-kb sequence consists of two simple head-to-tail pWCP units (I think this is what the authors are saying here) and whether their junction differs from an expected tandem duplication. The assembly and the four long reads make a biological multimer plausible, but they do not establish it uniquely. The host/lambda filter is useful for chimeras involving different source genomes, but it cannot remove a pWCP-pWCP ligation product or other same-reference artifact. Likewise, the absence of an internal adapter is reassuring but does not exclude every in-silico concatemer or “foldbacks” (a.k.a. duplicated inversions). ONT ligation libraries can generate chimeric molecules and substantially underrepresent small plasmids (Wick et al. 2021), while foldbacks and other long-read artifacts can lack detectable internal adapters, map with high confidence, and recur in multiple reads (Heinz et al. 2026). I’m not really an expert on this (and some of these artifacts could be explained or discounted) but I think these artifacts may be common enough that they should be considered and discussed in this context, especially due to the required read filtering. The result could alternatively be described as tandem or multimeric pWCP sequence consistent with a stable biological multimer, a rolling-circle replication intermediate or concatemer, a recombination product, or a technical artifact. Without stronger evidence, the data do not establish a stable discrete dimer, monomer/dimer coexistence, active replication, or a functional oriV. The categorical wording in the Title and Conclusion should perhaps therefore be moderated.

We thank the reviewer for these detailed comments and for drawing our attention to the different types of artifacts that may affect the interpretation of long-read sequencing data. We hope that we have correctly understood the reviewer's structural question. To clarify this point, the approximately 18.5-kb assembled sequence consists of two complete pWCP units arranged consecutively in the same orientation, in a simple head-to-tail tandem organization (Please see S3 Figure). The supporting long reads show the same direct tandem organization. The transition between successive units follows the expected pWCP sequence, with no additional sequence, deletion, or rearrangement at the junction relative to a simple tandem duplication. Thus, we do not observe a distinct junction sequence that would differentiate the assembled structure from two successive copies of pWCP. As pWCP is circular, the position at which each unit begins in a linear representation is arbitrary and does not correspond to a unique biological breakpoint.

We agree that this organization does not, by itself, establish a biological multimer and that potential sequencing artifacts must also be considered. Several types of chimeric or artifactual reads have been described in Oxford Nanopore sequencing data: (1) Ligation chimeras result from the physical joining of two distinct DNA molecules during library preparation (through adapters). (2) In silico chimeras can arise when two independent molecules pass successively through the same nanopore within a sufficiently short time interval for the ONT software to interpret them as a single read. (3) Foldback artifacts correspond to reads in which one portion aligns to a given locus in one orientation and another portion aligns to the same locus in the opposite orientation, producing a near-palindromic inverted duplication.

We first considered the possibility of a ligation chimera. In such cases, the artificial junction between the two molecules may be associated with the presence of an internal adapter sequence. We therefore searched for such sequences within the long reads supporting the multimeric pWCP structure, particularly around the junctions between repeated units. No internal adapter sequences were detected. This observation makes a ligation chimera less likely, although it does not formally exclude this possibility.

An *in silico* chimera involving two independent monomeric pWCP molecules remains theoretically possible. However, two conditions would have to be met for such an artifact to produce the direct, uninterrupted tandem organization observed here. First, the two molecules would need to enter the resulting read at matching, or nearly matching, positions along the pWCP sequence, so that their artificial concatenation would reproduce the expected transition between successive plasmid units without an additional sequence or a discontinuity. As shown in the newly added **S5 Table**, the pWCP reads have variable endpoints rather than consistently beginning and ending at the same positions. The formation of the observed tandem organization through the accidental concatenation of two independently sequenced monomeric molecules would therefore require a specific coincidence of their sequence coordinates. Second, these two plasmid molecules would have to pass successively through the same nanopore within a sufficiently short interval to be interpreted as a single read. We consider this scenario less likely in our dataset, which originates from a complex metagenomic sample containing several thousand reads, with pWCP representing only a limited fraction of the sequenced DNA. The probability that two independent monomeric pWCP molecules, beginning at the same relative position, would be sequenced immediately one after the other through the same pore would therefore be expected to be low. The recovery of several independent long reads displaying the same tandem organization also makes this explanation less parsimonious. Nevertheless, we acknowledge that an *in silico* chimera cannot be formally excluded based on the available data alone.

We also considered the foldback artifacts described by Heinz et al. In such artifacts, the two portions of a read align to the same region of the reference sequence but in opposite orientations, resulting in a near-palindromic structure or inverted duplication. This is not the configuration observed in our data. When aligned against a monomeric pWCP reference, the repeated units within the long reads align consecutively in the same orientation. They show no strand switch, inversion, or near-palindromic organization characteristic of a foldback artifact (see the corresponding figure from Heinz et al.). Such artifacts therefore do not appear to explain the reads supporting our assembly.

We nevertheless agree with the reviewer that our data do not allow us to demonstrate conclusively that the approximately 18.5-kb sequence represents a stable, discrete circular dimer. Our observations support a multimeric pWCP sequence composed of complete units arranged in a direct head-to-tail tandem orientation, but they do not formally distinguish a stable biological multimer from a non-biological concatemer, a rolling-circle replication intermediate, a recombination product, or an uncharacterized technical artifact.

We have therefore moderated the Title, Conclusion, and relevant sections of the manuscript so that this structure is no longer presented as categorical evidence for a stable dimer (Results, Lines 209- 212; 244; 271- 273 ; Discussion, Lines 377-379; 426- 439; Conclusion: Lines 540-542). We now state that the assembly and supporting long reads provide evidence for a tandem or multimeric organization of pWCP that is consistent with a biological origin, while explicitly acknowledging alternative interpretations and the limitations of the available sequencing data.

**Minor remaining comments**

I appreciate the additional analysis of possible WO connections, and am not requesting further analysis. However, I find the result still a bit difficult to follow. It would be helpful to have a visual representation or clearer description & quantitation of the read-level connections between contig 2 and WO regions, and whether any reads support a given chromosomal region without WO or with different WO integrations / structural arrangements.

We thank the reviewer for this comment. We realize that our response in the previous revision may have caused some confusion regarding the information that can actually be obtained from these reads. As described in the manuscript, the terminal region of contig_2 contains an Octomom-like region corresponding to part of the EAM (Eukaryotic Association Module). This type of phage-associated region has previously been described in *w*Pip reference genomes (Klasson et al., 2008; Bordenstein and Bordenstein, 2022) and is distinct from the regions containing the canonical WO phage structural genes. Thus, the end of contig_2 should not itself be interpreted as a resolved prophage integration boundary.

As shown in our previous response, some reads mapping to this terminal EAM/Octomom-like region also show partial alignments to other WO-rich contigs. We examined these reads more closely to determine whether they could provide additional information about the organization of these regions. However, the relevant reads are relatively short (mostly approximately 5–6 kb) and generally encompass only a few genes in this region. Moreover, their WO-associated portions are not uniquely assignable: the same reads can map to several WO-rich contigs and to different positions within these contigs because of the highly repetitive nature of these sequences (see IGV screenshot below). Consequently, these reads do not provide sufficient information to establish the synteny or structural relationships between the terminal region of contig_2 and the fragmented WO-rich contigs.

Regarding the reviewer's question about alternative prophage integrations or chromosomal regions without WO, our dataset does not provide the type of read-level information required to address this question. Such structural configurations would require reads spanning sufficiently informative chromosomal regions together with identifiable WO core regions, whereas, as noted in our previous response, we did not recover individual reads spanning clearly identifiable *Wolbachia* chromosomal core genes and extended prophage regions containing canonical WO structural genes. We therefore cannot use the present reads to distinguish alternative WO integrations, structural arrangements, or WO-free configurations.

We have revised the Results (new lines 298-305) to make these limitations clearer and to avoid implying that specific structural connections between contig_2 and individual WO-rich contigs were resolved. Given the short and non-specific nature of these alignments, we do not think that a schematic representation of contig-to-contig connections would accurately reflect the information contained in the data, as it could imply a level of structural resolution that these reads do not provide.

Finally, please check the contig counts in the Results: 24 initial contigs minus 10 excluded contigs appears to give 14 retained contigs, consistent with one core contig, one plasmid contig, and 12 smaller WO-rich contigs, rather than 15 retained contigs and 13 WO-rich contigs. But I may have just miscounted this.

We thank the reviewer for carefully checking these numbers and for pointing out this inconsistency. The reviewer is correct; this was an error on our part in the manuscript. We have corrected the contig counts throughout the Results section (new Lines 202-205) so that they now consistently report 14 retained contigs, comprising one core contig, one plasmid contig, and 12 WO-rich contigs. We sincerely appreciate the reviewer for noticing this mistake.

Response to Reviewers – FIRST REVIEW

*Our additional responses and changes made during the second revision are highlighted in purple. We have carefully addressed all comments from both rounds of review. To facilitate readability, we only display responses to comments with additional major modifications.*

Our additional responses and changes made during the second revision are highlighted in purple. We carefully addressed all comments from both rounds of review. To facilitate reading, we only display our response to comments with additional major modifications.

Reviewer #2:

**Major comments**

**WO phage integration, structural heterogeneity, potential activity**

• [296] “observed core genes flanking EAM regions in almost all contigs” — where are these core genes located on contig_2? This could provide evidence for the putative phage integration site(s).

We thank the reviewer for the comment. Here, the term “core genes” refer specifically to conserved WO phage core genes flanking EAM regions, as defined in a previous comparative genomic study based on an exhaustive analysis of WO prophages in *Wolbachia* (doi: 10.1371/journal.pgen.1010227). The positions of these phage-associated genes within contig_2 are already indicated slightly earlier in the manuscript. To further improve clarity and provide additional contextual information regarding neighboring genes, we also added the complete GFF annotation file together with Pfam annotations in S6 Table.

Additional response to the second-round review: In the present revision, we have further revised this section of the Results to clarify which WO-associated genes and regions are present in contig_2 and the smaller WO-rich contigs. We have also clarified that the available read alignments do not allow us to identify specific prophage integration sites or resolve the structural relationships between contig_2 and the smaller WO-rich contigs (new lines 298 -305).

• [301/315] The authors note that the *cif* and Octomom-like sequence at contig_2 edges strongly suggest a phage-associated integration region. More explicit read-level support would strengthen this interpretation: are there any reads mapping to the ends of contig 2 which span across this putative phage integration site? And/or do the ends of any phage contigs (especially the ones with more uniform coverage) map back to contig_2? This could also be done at an individual read level. The Flye —keep-haplotypes flag (or running a tool like Sniffles2) could also be helpful here.

We thank the reviewer for this insightful suggestion. Indeed, several long reads mapping to the terminal region of contig_2 also partially map to contig_10 and contig_22 (see IGV screenshot below, red rectangle), supporting the hypothesis that these regions likely correspond to prophage integration-associated regions. However, this point remains at the contig level. We did not recover any sufficiently individual long reads spanning both clearly identifiable *Wolbachia* chromosomal core genes within contig_2 and extended prophage regions containing canonical WO phage structural genes outside the EAM module. Consequently, although the observed read mappings support the presence of phage-associated regions connected to contig_2, the available data do not allow confident resolution of the exact structural organization or integration state of these prophage regions. We added these clarifications to the revised manuscript in order to strengthen and better contextualize the interpretation regarding putative prophage integration sites.

Additional response to the second-round review: Following the reviewer’s additional comments, we have re-examined the interpretation of these read alignments and clarified our description in the Results. The terminal Octomom-like region of contig_2 corresponds to part of the WO-associated EAM and should not itself be interpreted as a resolved prophage integration boundary. Although some reads mapping to this region also show partial alignments to contig_10, contig_22, and other WO-rich contigs, closer inspection revealed that these reads are relatively short (mostly 5–6 kb) and that their WO-associated portions can align to multiple contigs and positions within them. These non-specific alignments therefore do not establish particular contig-to-contig connections or allow us to resolve prophage integration sites, alternative WO arrangements, or WO-free configurations. We have revised the manuscript to make clear what these reads show and, importantly, what cannot be inferred from them (new lines 303 -305).

**Inference of MGE activity and mobilome dynamics**

The framing of an active, dynamic *Wolbachia* mobilome is plausible, but

[Title] I’m admittedly a bit hesitant about the word “dynamics” here, though it’s reasonable if the manuscript makes clear that some inferred dynamics come from structural heterogeneity rather than direct temporal observation.

We thank the reviewer for this thoughtful remark and agree that the term “dynamics” could imply direct temporal observations or experimentally demonstrated activity, which is beyond the scope of the current dataset. Accordingly, we revised the Title to better reflect that the inferred mobilome variability primarily arises from structural heterogeneity observed within the sequencing data.

The new title is now: “Extensive mobilome structural heterogeneity in *a widespread endosymbiont:* dimeric plasmids and highly fragmented prophages in *Wolbachia* from *Culex pipiens*”.

Additional response to the second-round review: In the present revision, we have further moderated the title to avoid implying that the observed pWCP tandem organization establishes the presence of a stable biological dimer. **The revised title is now: “Extensive mobilome heterogeneity in a widespread endosymbiont*:* a candidate dimeric plasmid and highly fragmented prophages in *Wolbachia* from *Culex pipiens* ”.**

**Minor general comments**

The introduction mentions small MGEs such as insertion sequences and transposons.

Given the nature of the long read data generated here, it might be interesting to briefly

comment on any observed IS/transposon heterogeneity or putative activity in the chromosome (other than what was found on WOPip5).

We thank the reviewer for this suggestion. Quantification analyses of transposase-associated regions across the *Wolbachia* genome were originally presented in Supplementary Figure 8. However, because one of the main conclusions of this study is that assembly reconstruction remains unreliable for several highly repetitive regions, particularly within the mobilome, we primarily focused our interpretation on coverage profiles and direct long-read analyses rather than on assembly-derived structural comparisons alone.

Additional response to the second-round review: we have now made the transposase-related results, previously presented only in S7 Fig, more explicit in the Results (new lines 334- 337).

The Discussion could briefly mention how newer ONT chemistry (R10.4.1), basecalling

(Dorado), and adaptive sampling approaches (to preferentially reject host & lambda

molecules) might improve future work on similar low-biomass, host-rich samples.

We thank the reviewer for this suggestion. We added a sentence in Methods, commenting on these topics.

“PCR amplification is often used to increase DNA yield when working with low-biomass samples but may introduce amplification biases and alter the relative representation of genomic sequence. To minimize these potential artefacts, a direct long read sequencing strategy was employed without prior whole genome amplification. Because the amount of ovarian DNA recovered from a single mosquito was below the recommended input for Nanopore library preparation (1µg genomic DNA), *lambda* phage DNA was added as carrier DNA to reach the minimum required DNA quantity (detailed in **S7** **Table**). Similar carrier DNA approaches have previously been proposed for ultra-low input sequencing applications to improve library preparation efficiency while preserving amplification free workflows [60]. Although carrier DNA may reduce the proportion of informative reads originating from the biological sample, it can subsequently be computationally removed from downstream analyses “.

The fast-evolving long-read sequencing technologies, such as the newest ONT chemistries and basecalling platforms—R10.4.1 flow cells and Dorado basecalling, compared to our current R9.4.1 flow cells and Guppy basecaller—may indeed enable much deeper sequencing of low-biomass samples in the near future, potentially revealing rare phage WO variants and other low-abundance genomic features. However, several challenges and potential disadvantages remain.The extremely large size of the Culex genome compared to *Wolbachia* genomes—a roughly 400-500-fold difference—poses a substantial obstacle. ONT experts at the London Calling 2022 event suggested that adaptive sampling, while promising for targeted enrichment, may lead to rapid exhaustion of sequencing pores when applied to such disproportionately large host genomes (personal communication to JR). This could result in dramatically reduced output, limiting the recovery of the target low-abundance sequences and this approach was therefore avoided in this context. The newer chemistries and basecallers, while improving raw read accuracy and throughput, can introduce biases in sequence representation, particularly for regions with high GC content, repetitive sequences, or modified bases. Moreover, while the theoretical yield per flow cell is higher, practical issues such as pore clogging, library preparation variability, and DNA degradation in low-biomass samples may still constrain effective sequencing depth.

Additional response to the second-round review: we briefly mentioned in the Discussion of the current version (new Lines 492-500) how newer ONT chemistry (R10.4.1), basecalling (Dorado), and adaptive sampling approaches might improve future work on similar low-biomass, host-rich samples while discussing challenges associated with these methods in this context.

**Minor line-by-line comments**

• Not sure if I’m interpreting this correctly, but could read 2 map well (anti-sense) to

palindrome 2 if intII was mobilized? It’s a bit hard to make sense of what might be happening here.

We thank the reviewer for this insightful observation. Indeed, the newly renamed read 15 (previously read 2) could potentially align to the Palindrome 2 configuration if the *intII* gene were present. Interestingly, the genes *mutL*, *intII*, and *rnhA* appear to be key variable elements distinguishing the different *cif*-associated configurations observed in our dataset. Similar patterns were recently described by Amoros et al. (2026), where the presence, absence, and organization of these genes contributed to the definition of distinct *cif* genomic islands.

Additional response to the second-round review: To make these configurations easier to interpret, we have now clarified in the Results the differences between the genomic regions flanking the two Type I *cif* palindromes (new lines 351- 356). We now describe these distinct genomic contexts explicitly in the manuscript to better explain the structural differences represented in Fig 5.B.
